# Prenatal dietary exposure to mixtures of chemicals is associated with allergy or respiratory diseases in children in the ELFE nationwide cohort

**DOI:** 10.1186/s12940-023-01046-y

**Published:** 2024-01-09

**Authors:** Manel Ghozal, Manik Kadawathagedara, Rosalie Delvert, Amandine Divaret-Chauveau, Chantal Raherison, Raphaëlle Varraso, Annabelle Bédard, Amélie Crépet, Véronique Sirot, Marie Aline Charles, Karine Adel-Patient, Blandine de Lauzon-Guillain

**Affiliations:** 1 Université Paris Cité and Université Sorbonne Paris Nord, Inserm, INRAE, Centre for Research in Epidemiology and StatisticS (CRESS) Equipe EAROH, Batiment Leriche, 16 avenue Paul Vaillant Couturier, Paris, Villejuif Cedex 94807 France; 2 Université Paris-Saclay, UVSQ, Université Paris-Sud, Inserm, Équipe d’Épidémiologie Respiratoire Intégrative, CESP, Villejuif, 94805 France; 3 Unité d’allergologie pédiatrique, Hôpital d’enfants, CHRU de Nancy, Vandoeuvre les Nancy, France; 4 EA 3450 DevAH, Faculté de Médecine, Université de Lorraine, Vandoeuvre les Nancy, France; 5 UMR 6249 Chrono-Environnement, Université de Bourgogne Franche Comté, Besançon, France; 6 Inserm, Team EPICENE, Bordeaux Population Health Research Center, UMR 1219, Bordeaux University, Bordeaux, France; 7 ANSES, French Agency for Food, Environmental and Occupational Health and Safety, Risk Assessment Department, Methodology and Studies Unit, Maisons-Alfort, France; 8 Université Paris-Saclay, CEA, INRAE, Département Médicaments et Technologies pour la Santé (MTS), Gif-sur-Yvette, France

**Keywords:** Food chemicals, Mixtures, Prenatal exposure, Allergy, Respiratory diseases, Birth cohort

## Abstract

**Introduction:**

Prenatal exposure to environmental chemicals may be associated with allergies later in life. We aimed to examine the association between prenatal dietary exposure to mixtures of chemicals and allergic or respiratory diseases up to age 5.5 y.

**Methods:**

We included 11,638 mother-child pairs from the French “Étude Longitudinale Française depuis l’Enfance” (ELFE) cohort. Maternal dietary exposure during pregnancy to eight mixtures of chemicals was previously assessed. Allergic and respiratory diseases (eczema, food allergy, wheezing and asthma) were reported by parents between birth and age 5.5 years. Associations were evaluated with adjusted logistic regressions. Results are expressed as odds ratio (OR[95%CI]) for a variation of one SD increase in mixture pattern.

**Results:**

Maternal dietary exposure to a mixture composed mainly of trace elements, furans and polycyclic aromatic hydrocarbons (PAHs) was positively associated with the risk of eczema (1.10 [1.05; 1.15]), this association was consistent across sensitivity analyses. Dietary exposure to one mixture of pesticides was positively associated with the risk of food allergy (1.10 [1.02; 1.18]), whereas the exposure to another mixture of pesticides was positively but slightly related to the risk of wheezing (1.05 [1.01; 1.08]). This last association was not found in all sensitivity analyses. Dietary exposure to a mixture composed by perfluoroalkyl acids, PAHs and trace elements was negatively associated with the risk of asthma (0.89 [0.80; 0.99]), this association was consistent across sensitivity analyses, except the complete-case analysis.

**Conclusion:**

Whereas few individual chemicals were related to the risk of allergic and respiratory diseases, some consistent associations were found between prenatal dietary exposure to some mixtures of chemicals and the risk of allergic or respiratory diseases. The positive association between trace elements, furans and PAHs and the risk of eczema, and that between pesticides mixtures and food allergy need to be confirmed in other studies. Conversely, the negative association between perfluoroalkyl acids, PAHs and trace elements and the risk of asthma need to be further explored.

**Supplementary Information:**

The online version contains supplementary material available at 10.1186/s12940-023-01046-y.

## Introduction

Environmental chemicals can alter different components of the intestinal homeostasis (epithelial barrier, microbiota, immune system), and thus, lead to health effects. Exposure to environmental chemicals during the perinatal period can induce dysbiosis of the microbiota and/or an alteration of intestinal structure and permeability, and ultimately to intestinal inflammation which can lead to chronic diseases [24, 55]. However, the amplitude and health effects in humans in the short, medium and long term of this pre- and perinatal exposure remain to be elucidated, particularly in the context of complex mixtures [52].

Prenatal exposure to environmental chemicals might play a role in shaping the immune system and epithelial barriers, predisposing to the development of allergic or respiratory diseases later in life [2, 20]. Under the concept of the ‘epithelial barrier hypothesis’, several studies showed a harmful effect of early life exposure to toxic agents is likely to cause barrier damages affecting the integrity of the epithelial barrier which when occurring in tissues such as skin and mucosal sites leads to type 2 inflammation and development of allergic diseases [14, 47]. Furthermore, exposure to environmental chemicals has been associated with an oxidative stress leading to impairment of lipids and protein in the epidermis and thus to skin barrier dysfunction that is implicated in the development and aggravation of atopic dermatitis [1].

Some epidemiologic studies have reported an increased risk of allergies or asthma in children born to mothers exposed to some environmental chemicals during pregnancy, such as pesticides [13], perfluoroalkyl acids (PFAAs) [44], phthalates [63], trace elements (TE) [50], polycyclic aromatic hydrocarbons (PAHs) [34], bisphenol A (BPA) [68], brominated flame retardants (BFRs) [42] or polychlorobiphenyls (PCBs) [46]. Inversely, other studies found a decreased risk of allergy or wheezing related to the prenatal exposure to some PFAAS [2], pesticides [41], BFRs [43], and BPA [19]. Finally, other studies did not highlight any association [7, 9].

Nevertheless, these studies considered chemicals separately, while individuals are exposed simultaneously to a plethora of substances. Moreover, in Europe, human monitoring for several environmental chemicals in the research project HBM4EU evidenced higher levels of exposure than health based guidance values, which further highlighted concerns about effects on health of such chemical mixtures [22, 38]. Our diet is an important vector of multiple exposure to chemicals, and food is the main source of exposure to a large set of environmental chemicals [45].

Some studies have explored the effect of multiple exposure to chemicals during pregnancy on allergies and asthma in children. For instance, two studies from the Helix [25] and the CHAMACOS [8] cohorts have explored the effect of prenatal exposure to multiple environmental and chemical factors on childhood allergic and respiratory outcomes and have identified associations between few chemicals and asthma or respiratory allergies. In the INUENDO birth cohort, principal components analysis (PCA) was applied to reduce prenatal exposure to 16 chemicals in five principal components (PCs), but limited evidence of an association with eczema and asthma were reported [58]. However, even though chemicals were considered simultaneously in these studies, they fail to consider potential interactions between substances and the resulting mixture effects.

Two other studies used the Bayesian Kernel Machine Regression (BKMR) method to explore the link between the combined effect of prenatal exposures to chemicals on allergic outcomes in children. They reported mixture effects of BPA and phthalates on atopic dermatitis [36] and metals on allergic rhinitis [53].

Besides the limited number of studies exploring the effect of prenatal exposure to mixtures of chemicals on allergies, the studied mixtures are not composed of the same chemicals. Thus, comparison between these studies remain very complex. In the Eden mother-child cohort, eight mixtures of chemicals were identified from maternal dietary exposure in pregnancy using sparse non-negative matrix underapproximation (SNMU) [62]. We previously reported a positive association between prenatal dietary exposure to a mixture composed mainly by trace elements, furans and PAHs and allergic rhinitis up to 8 years [21].

Thus, the aim of the present study was to replicate our previous analysis on associations between prenatal dietary exposure to eight mixtures of chemicals and the risk of allergic or respiratory diseases up to 5.5 years, in a larger population.

## Methods

### Study population

The “Étude Longitudinale Française depuis l’Enfance” (ELFE) study is the first French nationwide birth cohort. This study enrolled 18,329 children and their families at birth in a random sample of 349 maternity units during 2011. Recruitments were conducted in four waves of 4–8 days each, one for each season of the year. The study included children from single or twin’s pregnancy, born after 33 weeks of gestation, to mothers aged 18 years or older and who were not planning to move outside of metropolitan France in the next 3 years [16].

Enrolled mothers signed a written consent for their own and participation of their child. Fathers present at inclusion signed the consent form for the participation of their child or were informed of their right to object.

The ELFE study received approvals from the Advisory Committee for the Treatment of Information on Health Research (Comité Consultatif sur le Traitement des Informations pour la Recherche en Santé), the National Agency Regulating Data Protection (Commission Nationale Informatique et Libertés), and the National Statistics Council (Conseil National de l’Information Statistique).

### Prenatal exposure to food chemicals

Assessment of maternal exposure to food chemicals during the last 3 months of pregnancy has been previously described [62]. Briefly, maternal diet during the last trimester of pregnancy was assessed at delivery using a validated semi-quantitative food frequency questionnaire (FFQ) [29]. Then, food intakes were combined to the concentrations of chemicals assessed in food items within the second French Total Diet Study (TDS 2) [15, 57]. In our study, the lower bound (LB) scenario was applied. According to this scenario, values below the limit of detection were replaced by a “zero”, and values above the limit of detection but below the limit of quantification were assigned to the limit of detection values. Maternal exposure to mixtures of chemicals was previously determined using the sparse non-negative matrix under-approximation (SNMU) method. This method of dimension reduction is specific to non-negative matrices, and thus, is suitable for nutritional exposures data composed of positive values and with many zero values [6]. Applying this method, the maternal chemical exposures of the studied population can be expressed as a matrix E of dimension (PxN) where P is the number of substances and N of individuals. E is factorized by two low ranked (PxK) and (KxN) non-negative matrices of K factorial dimensions designating the number of exposure systems [62, 67]. To choose the optimal K value, SNMU is run for different values of K and the residual sum of squares were minimized as explained in Traoré. Since units of chemicals concentration are not the same between all chemicals, concentrations were reduced to avoid scaling effect.

Eight mixtures were identified on the basis of 210 detected chemicals out of 441 substances analyzed in TDS2, including: 73 pesticides, 21 trace elements, 20 PAHs, 18 PCBs, 17 dioxins and furans, 18 mycotoxins, 14 BFRs, 12 PFAAs, 11 phytoestrogens, 4 food additives, acrylamide and bisphenol A (BPA) (Supplementary Table 1). Mixtures were named according to substances contributing the most in their composition (Table 1) [62]:

- *TE-F-PAH:* 12 trace elements - 2 dioxins - 5 furans - pyrene.
- *PCB-BFR-Aso-MeHg:* 9 PCBs - 7 BFRs - 2 trace elements – 2 furans
- *Pest-1:* 3 carbamates - 4 organophosphorus pesticides (OPs) - 2 dicarboximides - 3 benzoylureas - 7 other pesticides - patulin
- *Pest-3:* 3 pyrethroids - 4 triazoles - 13 other pesticides
- *PFAA-Ge-Li:* 6 PFAAs - 4 trace elements - 6 pesticides – 4 mycotoxins
- *Pest-2:* 2 pyrethroids - 2 strobilurins - 4 OPs - 2 dicarboximides - 3 triazoles - 7 other pesticides
- *Mixt3:* 5 PFAAs - 12 PAHs - 2 trace elements - 1 BFRs
- *Mixt4:* 19 pesticides – trace element tin

**Table 1:** Description of the eight dietary chemicals mixtures identified from ELFE during pregnancy, and their 20 main contributors. The percentage of explained variance by each mixture is presented in parentheses

NMU 1		NMU 2		NMU 3		NMU 4		NMU 5		NMU 6		NMU 7		NMU 8	
TE-F-PAH (31.4%)	% Subst	**PCB-BFR-Aso-MeHg (10.7%)**	% Subst	**Pest-1 (12.9%)**	% Subst	**Pest-3 (6.9%)**	% Subst	**PFAA-Ge-Li (16.0%)**	% Subst	**Pest-2 (7.5%)**	% Subst	**Mixt-3 (4.4%)**	% Subst	**Mixt-4 (10.2%)**	% Subst
CrVI	2.3	PCB_123	3.1	Propargite	5.9	Myclobutanil	5.3	PFHxA	11.5	Bupirimate	7.9	PFTeDA	6.4	Chlorothalonil	10.9
CrIII	2.2	PBDE47	3.0	Thiabendazole	5.8	Fenhexamid	5.2	PFHpA	11.5	Dichlorvos	7.9	BkF	6.4	Diethofencarb	10.9
Pb	2.2	PBDE100	3.0	Pyrimicarb	5.7	Triadimenol	5.1	PFHxS	11.4	Endosulfan	7.8	BbF	6.4	Carbofuran	10.9
Co	2.2	PCB_52	3.0	Phosalone	5.7	Etofenprox	5.0	PFBS	11.4	Acrinathrin	7.8	BjF	6.2	Tetradifon	10.8
Ba	2.1	PCB_101	3.0	Diphenylamine	5.7	Trifloxystrobin	5.0	PFOA	8.6	Kresoxim_methyl	7.6	DbaiP	6.1	Pyriproxyfen	10.8
Te	2.1	PBDE154	3.0	Diflubenzuron	5.7	Quinoxyfen	5.0	Carbendazim	4.5	Azoxystrobin	7.0	PFDoA	5.4	Chlortal_dimethyl	8.9
V	2.1	PCB_77	3.0	Folpet	5.7	Spiroxamine	5.0	CrVI	2.4	Penconazole	5.2	CHR	5.1	Procymidone	6.7
Ni	2.0	PCB_153	2.9	Azinphos_methyl	5.7	Cyfluthrin	5.0	FB 1	2.0	Boscalid	4.7	BaA	4.2	Pyrimethanil	4.6
Asi	1.9	PBB153	2.9	Captan	5.7	Tetraconazole	5.0	OTA	1.7	Tebuconazole	4.5	DbaeP	4.1	Metalaxyl_M	4.2
Sb	1.9	PCB_105	2.9	Ethoxyquin	5.7	Tebufenpyrad	5.0	FB 2	1.7	Dimethoate	4.4	BaP	3.9	Iprodione	3.3
HCDF_123678	1.9	PBDE28	2.9	Triflumuron	5.7	Methomyl	4.5	Li	1.5	Fenbuconazole	4.4	Ag	3.5	Bifenthrin	2.5
PY	1.9	PBB52	2.8	Tebufenozid	5.7	Teflubenzuron	4.3	Phenylphenol	1.4	Mepanipyrim	4.1	IP	3.2	Sulfur	2.4
Al	1.8	PCB_157	2.8	Carbendazim	5.5	Pyrimethanil	4.1	Sn	1.2	Procymidone	3.8	DBahA	2.8	Cyproconazole	2.0
HCDF_123478	1.8	TCDF_2378	2.8	Phosmet	3.3	Cyprodinyl	3.9	Pyriproxyfen	1.2	Phosmet	2.9	HBCDbeta	2.3	Sn	1.3
HCDF_1234789	1.8	PCB_167	2.7	Pat	2.8	Bifenthrin	3.8	PFOS	1.2	Chlorpyrifos_ethyl	2.6	PFDA	2.3	Cyprodinyl	1.3
OCDF	1.8	PBB101	2.7	Fludioxonyl	2.3	Mepanipyrim	3.7	Ge	1.1	Metalaxyl_M	2.5	DbalP	2.2	Lambda_Cyhalothrin	1.1
Li	1.7	PCB_114	2.6	Chlorpyrifos_ethyl	2.1	Metalaxyl_M	3.5	Tetradifon	1.0	Lambda_Cyhalothrin	2.4	PFOS	2.1	Fludioxonyl	1.1
HCDF_123789	1.7	PCDF_12378	2.6	Teflubenzuron	1.6	Penconazole	3.0	DON15	1.0	Cyprodinyl	2.0	BcFL	2.0	Secoisolariciresino	0.7
HCDD_123678	1.7	PCB_138	2.5	Boscalid	1.5	Boscalid	2.3	Lindane	1.0	Fludioxonyl	1.5	Aso	1.8	Flutriafol	0.7
HCDD_123789	1.7	PCB_189	2.5	Methomyl	1.2	Iprodione	1.9	Sulfur	0.9	Iprodione	1.2	PFNA	1.7	Imidacloprid	0.7

Each individual had a score on each mixture, representing the weights of adherence of his/her exposure to each mixture*.* Scores of exposures were divided by the standard deviation (SD) to allow estimation of odds ratio (OR) for a variation of one SD increase in the mixture pattern and facilitate comparison of effect size across mixtures*.*

### Allergic and respiratory events

Respiratory and allergic events were reported by parents during phone interviews at age 2 months, 1, 2, 3.5, and 5.5 years. Children were considered as ever having wheezing from birth to 5.5 years if parents reported at least once that the child had wheezing in the chest at any of the follow-ups. Similarly, children were considered as ever having eczema from birth to 5.5 years if parents reported at least once that the child had eczema symptoms (itchy rash) at any of the follow-ups. Children were defined as having current asthma, according to the ISAAC definition [5], if parents reported at least once a medical diagnosis of asthma combined with (1) current wheezing or (2) current use of specific asthma medication, at the 2-, 3.5-, or 5.5-year follow-ups. Because food allergies were not collected at the 1-year follow up, children were considered as ever having food allergies if parents reported a medical diagnosis of cow’s milk protein allergy at the 2-month follow-up or at least once medical advice to avoid certain foods due to an allergy at the 2-, 3.5-, or 5.5-year follow-ups.

### Study sample

**Fig. 1:**
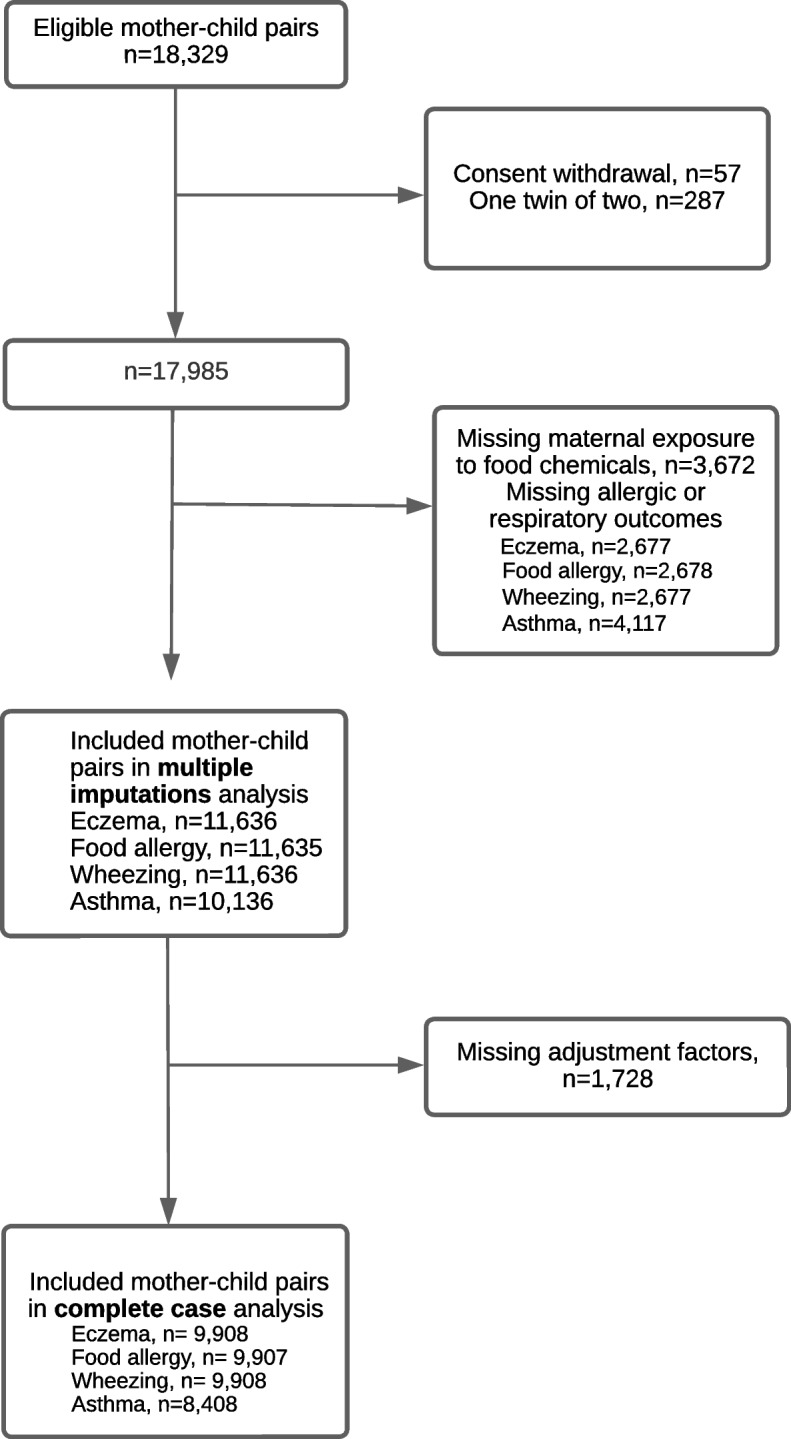
Flow chart of the study population

Eighteen thousand three hundred twenty-nine mother-child dyads were included in the ELFE study. Children whose parents withdrew consent (*n* = 57) were excluded from the study. We randomly selected one twin of two (*n* = 287) to avoid family clusters. We excluded children with missing data on maternal exposure to food chemicals (*n* = 3672) and those with missing allergic or respiratory events, leading to a main sample of 11,636 children (*n* = 11,636 for eczema and wheezing, *n* = 11,635 for food allergy, and *n* = 10,136 for current asthma, Fig. 1). For the complete-case analyses, we excluded dyads with missing values on confounding factors (*n* = 1728).

### Statistical analysis

Included and excluded samples were compared by chi-square and Student t tests for categorical and continuous variables, respectively. Firstly, the associations between prenatal exposure to the eight food chemicals mixtures and the risk of allergy or respiratory diseases were examined using adjusted logistic regression considering all the 8 mixtures simultaneously. Results are expressed as odds ratio (OR[95%CI]) for a variation of one SD increase in mixture pattern.

Potential confounding factors were first identified based on literature and then selected using the directed acyclic graphs (DAG) method [56] (Supplementary Fig. 1). All regression models were then adjusted for maternal age, education level, employment status, household income, number of older children in the household, maternal smoking during pregnancy, migration status, maternal diet quality through a score based on the probability of adequate nutrients intake (PANDiet score) [10], maternal rural residence, maternal region of residence and family history of allergies. Because they are related to allergies but not in the causal pathway from food chemical exposure to allergies, we decided to adjust also for child’s sex, and mode of delivery. We also adjusted for variables related to study design (maternity size and study wave).

To deal with missing values on confounding factors, principal analyses were conducted using multiple imputations models. Data were assumed to be missing at random, and 5 independent datasets were generated with the fully conditional specification method (MI procedure, FCS statement, NIMPUTE option), then pooled effect estimates were calculated (SAS MIANALYSE procedure). Continuous variables were imputed with predictive mean matching, and logistic regressions were used for categorical variables (Supplementary Table 2).

We also conducted some sensitivity analyses. To account for selection and attrition bias, we performed a sensitivity analysis with weighted data. Weighting was calculated by considering the inclusion procedure and biases related to non-consent [28] and also included calibration on margins from the state register’s statistical data and the 2010 French National Perinatal study [12] on the following variables: age, region, marital status, migration status, level of education, and primiparity. A second sensitivity analysis was conducted on a complete-case sample. To account for a potential overestimation of eczema or wheezing cases due to a single parental report of symptoms, a sensitivity analysis was conducted by excluding individuals with only one parental report of food allergy, eczema, asthma or wheezing.

Additionally, 210 food chemicals were considered individually to assess associations with allergic or respiratory events. Adjusted logistic regression models were used and a *p*-value *<* 0.05 was considered statistically significant. To deal with multiple testing issues, we used the False Discovery Rate procedure (SAS, PROC Multtest) and a q-value *<* 0.1 was considered statistically significant. Daily exposure to each food chemical was log-transformed to account for their skewed distribution and then standardized to allow comparison of effect size across chemicals. For 98 chemicals with very low proportion of exposed subjects, we performed categorization as follows: low exposure (1st tercile) and medium/high exposure (2nd / 3rd terciles). Analyses were conducted using multiple imputations models.

All analyses were conducted with SAS v9.4 (SAS Institute, Cary, NC, USA).

## Results

### Population description

Compared to excluded mothers, mothers included in at least one model (*n* = 11,636) were older (31.0 vs 30.3 years), more educated (21.0% vs 17.6% with ≥5-y university degree), with a higher household income (1661 vs 1513 €/month/consumption unit) and less likely to be immigrants (8.2% vs 18.2%) or smoker during pregnancy (19.3% vs 22.6%). No difference between included and excluded children was reported regarding sex, mode of delivery or older siblings, but included children were more likely to have family history of allergy (56% vs 53%).

In the included study sample, 44% of mothers were primiparous, 51% of children were male and 17.6% were born by cesarean section. In this population, the prevalence of any wheezing, asthma, eczema and food allergy up to 5.5 years were 40, 8, 48, and 6%, respectively (Table 2).

**Table 2:** Caracteristics of the main sample (*n* = 11,636)

Maternal characteristics		
Age at delivery, mean (SD)		31 (4.86)
Education level, % (n)		
	Up to lower secondary	3.3% (419)
	Upper secondary	32.8% (4179)
	Intermediate	24.2% (3074)
	3-y university degree	18.7% (2383)
	At least 5-y university degree	21% (2669)
Employed during pregnancy, % (n)		74.2% (9737)
Family income, (€/month/consumption unit), mean (SD)		1661 (930)
Migration status, % (n)		
	Immigrant	8.2% (1071)
	Descendant of at least one immigrant	10.1% (1314)
	Rest of population	81.7% (10682)
Smoking during pregnancy, % (n)		19.3% (2556)
Older siblings, % (n)		
	No older siblings	43.6% (5721)
	One older sibling 37.9% (4974)	37.9% (4974)
	At least two older siblings	18.6% (2440)
Maternal diet quality (PANDiet-G, 0–100 score), mean (SD)		55.7 (9.1)
Living in a rural area (< 2000 inhabitants), n (%)		23.4% (3133)
**Child and family characteristics**		
Boys, % (n)		51% (6820)
C-section, % (n)		17.6% (2333)
Family history of allergy, % (n)		50.3% (6539)
**Respiratory and allergic diseases, % (n)**		
Wheezing from 0 to 5.5 years		40.2% (5377)
Current asthma (ISAAC^a^ definition) from 0 to 5.5 years		7.7% (890)
Eczema from 0 to 5.5 years		47.8% (6383)
Food allergy from 0 to 5.5 years		6.4% (847)
**Maternal dietary exposure to mixtures of chemicals, mean (SD)**		
TE-F-PAH		1.55 (0.6)
Mixt-3		0.18 (0.3)
Pest-1		0.65 (1.0)
Pest-3		0.33 (0.9)
PCB-BFR-Aso-MeHg		0.50 (0.6)
Pest-2		0.36 (0.8)
Mixt-4		0.53 (0.9)
PFAA-Ge-Li		0.84 (1.0)

^a^*ISAAC* International Study of Asthma and Allergies in Childhood, *SD* standard deviation

### Eczema

Prenatal dietary exposure to the mixture TE-F-PAH, composed mostly by trace elements, furans and PAHs, was associated with a higher risk of eczema (OR, CI 95% 1.10 [1.05; 1.15] for 1 SD increase in mixture score) (Table 3). Similar findings were found in complete-case analysis (Table 4) in the weighted model (Table 5) and in the model excluding cases with only one report of eczema during follow up (Supplementary Table 3).

**Table 3:** Associations between prenatal dietary exposure to chemicals mixtures and the risk of allergic or respiratory diseases in early childhood (analysis with multiple imputations)

Mixture name	OR [95% CI]			
	Eczema (*n* = 11,636)	Food allergy (*n* = 11,635)	Wheezing (*n* = 11,636)	Asthma (*n* = 10,136)
TE-F-PAH	1.10 [1.05; 1.15]	0.97 [0.89; 1.07]	1.00 [0.96; 1.05]	0.98 [0.89; 1.08]
Mixt-3	0.98 [0.95; 1.02]	0.96 [0.88; 1.05]	1.00 [0.96; 1.04]	0.89 [0.80; 0.99]
Pest-1	1.00 [0.96; 1.04]	1.10 [1.02; 1.18]	0.99 [0.95; 1.03]	0.94 [0.87; 1.02]
Pest-3	1.00 [0.97; 1.04]	1.04 [0.96; 1.12]	0.99 [0.95; 1.03]	1.03 [0.95; 1.11]
PCB-BFR-Aso-MeHg	0.98 [0.94; 1.02]	1.03 [0.95; 1.11]	0.97 [0.93; 1.02]	0.94 [0.86; 1.03]
Pest-2	0.97 [0.93; 1.01]	0.96 [0.88; 1.04]	1.01 [0.97; 1.06]	0.96 [0.89; 1.05]
Mixt-4	0.98 [0.95; 1.02]	0.99 [0.92; 1.07]	1.05 [1.01; 1.08]	0.94 [0.87; 1.01]
PFAA-Ge-Li	1.00 [0.97; 1.04]	0.97 [0.90; 1.04]	1.02 [0.98; 1.05]	1.05 [0.98; 1.13]

Values are odds ratios [95% CI] for a variation of one standard deviation (SD) from logistic regression models including the eight mixtures simultaneously and adjusted for maternal characteristics (age, education level, migration status, employment, household income, rural residence, region of residence, number of older children in the household, smoking during pregnancy, diet quality), child and birth characteristics (sex, mode of delivery, family history of allergies), and variables related to study design (maternity size and recruitment wave). Abbreviations: *TE-F-PAH* trace elements-furans-polycyclic aromatic hydrocarbons, *PCB-BFR-Aso-MeHg* polychlorobiphenyls-brominated flame retardants-organic arsenic-methylmercury, Pest-1, pesticides-1; Pest-2, pesticides-2; Pest-3, pesticides-3; PFAA-Ge-Li, perfluoroalkyl acids-germanium-lithium; Mixt-3, mixture-3; Mixt-4, mixture-4

**Table 4:** Associations between prenatal dietary exposure to chemicals mixtures and the risk of allergic or respiratory diseases in early childhood (complete-case sample)

Mixture name	OR [95% CI]			
	Eczema (*n* = 9908)	Food allergy (*n* = 9907)	Wheezing (*n* = 9908)	Asthma (*n* = 8908)
TE-F-PAH	1.09 [1.04; 1.14]	0.97 [0.88; 1.07]	1.01 [0.96; 1.06]	0.99 [0.90; 1.10]
Mixt-3	0.99 [0.96; 1.04]	0.97 [0.88; 1.06]	0.99 [0.95; 1.04]	0.90 [0.81; 1.01]
Pest-1	0.99 [0.95; 1.03]	1.08 [1.00; 1.16]	0.98 [0.94; 1.02]	0.95 [0.87; 1.04]
Pest-3	1.02 [0.97; 1.06]	1.06 [0.98; 1.15]	1.00 [0.95; 1.04]	1.04 [0.95; 1.13]
PCB-BFR-Aso-MeHg	0.98 [0.94; 1.02]	1.01 [0.93; 1.10]	0.97 [0.93; 1.02]	0.96 [0.87; 1.05]
Pest-2	0.97 [0.93; 1.01]	0.96 [0.88; 1.05]	1.01 [0.97; 1.05]	0.95 [0.87; 1.04]
Mixt-4	0.98 [0.94; 1.02]	1.00 [0.92; 1.08]	1.05 [1.01; 1.09]	0.94 [0.86; 1.01]
PFAA-Ge-Li	1.00 [0.96; 1.04]	0.96 [0.89; 1.04]	1.01 [0.97; 1.05]	1.05 [0.98; 1.13]

Values are odds ratios [95% CI] from logistic regression models including the eight mixtures simultaneously and adjusted for maternal characteristics (age, education level, migration status, employment, household income, rural residence, region of residence, number of older children in the household, smoking during pregnancy, diet quality), child and birth characteristics (sex, mode of delivery, family history of allergies), and variables related to study design (maternity size and recruitment wave). Abbreviations: *TE-F-PAH* trace elements-furans-polycyclic aromatic hydrocarbons, *PCB-BFR-Aso-MeHg* polychlorobiphenyls-brominated flame retardants-organic arsenic-methylmercury; Pest-1, pesticides-1; Pest-2, pesticides-2; Pest-3, pesticides-3; PFAA-Ge-Li, perfluoroalkyl acids-germanium-lithium; Mixt-3, mixture-3; Mixt-4, mixture-4

**Table 5:** Weighted associations between prenatal dietary exposure to chemicals mixtures and the risk of allergic or respiratory diseases in early childhood

Mixture name	OR [95% CI]			
	Eczema (*n* = 11,636)	Food allergy (*n* = 11,635)	Wheezing (*n* = 11,636)	Asthma (*n* = 10,136)
TE-F-PAH	1.07 [1.02; 1.14]	1.02 [0.92; 1.14]	1.01 [0.95; 1.07]	0.98 [0.87; 1.11]
Mixt-3	0.99 [0.94; 1.03]	0.97 [0.86; 1.10]	1.00 [0.95; 1.05]	0.89 [0.80; 0.99]
Pest-1	1.01 [0.96; 1.06]	1.15 [1.06; 1.24]	0.98 [0.94; 1.03]	0.95 [0.86; 1.05]
Pest-3	1.01 [0.96; 1.06]	1.03 [0.95; 1.13]	1.00 [0.95; 1.06]	1.02 [0.92; 1.12]
PCB-BFR-Aso-MeHg	0.98 [0.94; 1.03]	1.03 [0.94; 1.14]	0.97 [0.92; 1.02]	0.88 [0.79; 0.98]
Pest-2	0.94 [0.89; 0.99]	0.93 [0.86; 1.02]	1.03 [0.98; 1.08]	0.99 [0.91; 1.09]
Mixt-4	0.97 [0.93; 1.02]	1.00 [0.90; 1.11]	1.04 [1.00; 1.09]	0.94 [0.86; 1.03]
PFAA-Ge-Li	1.00 [0.96; 1.05]	0.99 [0.91; 1.08]	1.03 [0.98; 1.08]	1.09 [0.99; 1.21]

Values are odds ratios [95% CI] from logistic regression models including the eight mixtures simultaneously and adjusted for maternal characteristics (age, education level, migration status, employment, household income, rural residence, region of residence, number of older children in the household, smoking during pregnancy, diet quality), child and birth characteristics (sex, mode of delivery, family history of allergies), and variables related to study design (maternity size and recruitment wave). Abbreviations: *TE-F-PAH* trace elements-furans-polycyclic aromatic hydrocarbons, *PCB-BFR-Aso-MeHg* polychlorobiphenyls-brominated flame retardants-organic arsenic-methylmercury; Pest-1, pesticides-1; Pest-2, pesticides-2; Pest-3, pesticides-3; PFAA-Ge-Li, perfluoroalkyl acids-germanium-lithium; Mixt-3, mixture-3; Mixt-4, mixture-4

When considered individually, prenatal dietary exposure to 75 chemicals, including 15 trace elements, 9 furans, and 15 PAHs, but also chemicals from other groups (4 BFRs, 7 dioxins, 7 mycotoxins, 14 BCBs, 2 pesticides) was associated with a higher risk of eczema (Supplementary Fig. 2 and supplementary Table 4). After multiple testing correction, all these associations remained significant (Supplementary Table 4).

### Food allergy

Prenatal dietary exposure to the mixture Pest-1, composed mainly by pesticides, was positively associated with the risk of food allergy (OR, CI 95% 1.10 [1.02; 1.18] (Table 3). Similar findings were found in the weighted model (Table 5) and in the model excluding cases with only one report of food allergy during follow up (Supplementary Table 3) but did not reach significance in complete-case analysis (Table 4).

When considered individually, prenatal dietary exposure to 4 chemicals was associated with a higher risk of food allergy (3 trace elements, and pesticide Chlorpyrifos-ethyl) (Supplementary Fig. 3 and Supplementary Table 5). Inversely, prenatal dietary exposure to 10 chemicals was associated with a lower risk of food allergy (2 PFAAs, 3 mycotoxins, 4 PAHs and pesticide carbaryl) (Supplementary Fig. 3).

After correction for multiple testing, associations with mycotoxins, pesticide carbaryl and trace element strontium remained significant (Supplementary Table 5).

### Wheezing

Prenatal dietary exposure to the mixture Mixt-4, composed mainly by pesticides, was positively associated with the risk of wheezing (OR, CI 95% 1.05 [1.01; 1.08]) (Table 3). Similar findings were reported in the complete-case analysis (Table 4) but did not reach significance in the weighted model (Table 5) nor in the model excluding cases with only one report of wheezing during follow up (Supplementary Table 3).

When considered individually, prenatal dietary exposure to 16 chemicals, including 15 pesticides was associated with a higher risk of wheezing, while PFHpA was negatively associated (Supplementary Fig. 4 and Supplementary Table 6). However, none of these associations remained significant after correction for multiple testing (Supplementary Table 6).

### Asthma

Prenatal dietary exposure to the mixture Mixt-3, composed mainly by PFAAs, PAHs and trace elements, was negatively associated with the risk of asthma (OR, CI 95% 0.89 [0.80; 0.99]) (Table 3). Similar findings were reported in the weighted model (Table 5) and in the model excluding cases with only one report of asthma during follow up (Supplementary Table 3), but did not reach significance in the complete-case analysis (Table 4).

When considered individually, prenatal dietary exposure to 28 chemicals, including 10 PAHs and PFHpA, but also 16 pesticides and mycotoxin DON15, was associated with a lower risk of current asthma (Supplementary Fig. 5 and Supplementary Table 7). After correction for multiple testing, 24 associations remained significant (Supplementary Table 7).

## Discussion

In this study, we explored the association between maternal dietary exposure to eight mixtures of chemicals and allergic or respiratory events up to 5.5 years in a large birth cohort. Higher risk of eczema was consistently observed in children prenatally exposed to a mixture of PAHs, furans and trace elements, while higher risk of food allergy was consistently observed among children with higher prenatal exposure to a mixture of pesticides. Conversely, a mixture of PFAAS, PAHs and trace element was negatively associated with the risk of asthma.

In the literature, studies on associations between exposure to chemicals and health outcomes mainly considered chemicals individually. This approach leads to results that are easy to represent, to interpret and to compare with other studies. However, it has some limitations. First, numerous of these substances are simultaneously present in the same food items resulting in collinearity between the studied exposures, which leads to reduced precision in the estimated coefficient [39]. Second, single exposure hypothesis is not representative of the reality of population’s exposure to numerous chemicals in diet. Co-exposure to environmental chemicals was associated with interactions between chemicals, leading to synergistic or antagonistic effects [11]. To deal with this issue, in the present study, we analyzed maternal exposure to food chemicals as mixtures to explore relations between co-exposure to food chemicals and allergic and respiratory events in childhood. One individual chemical can contribute to different mixtures, but to a greater or lesser extent.

A positive association was found between maternal dietary exposure to a mixture composed mainly by trace elements, furans and PAHs (TE-F-PAH mixture) and the risk of eczema in children. The association was quite weak but consistent across sensitivity analyses. Several chemicals belonging to these three groups of chemicals, but also to other groups (dioxins, PCBs and mycotoxins), were individually associated with eczema. Positive association between prenatal exposure to metals and the occurrence, the severity or the duration of atopic dermatitis in childhood have been reported previously [32, 33, 35, 36, 50, 64]. However, a recent meta-analysis concluded to limited evidence of an association between pre and postnatal exposure to heavy metals and allergies (asthma, atopic dermatitis, eczema and wheeze) in childhood (Wang, Yin et al. 2022). In the LaSalle EUC Retrospective Mortality Cohort, prenatal exposure to PCBs was positively associated with the risk of eczema up to 10 years [46]. In the EDEN mother-child cohort, prenatal dietary exposure to the mixture TE-F-PAH was associated with higher risk of allergic rhinitis but not with eczema, and none of the other mixtures was associated with allergic or respiratory diseases up to 8 years [21]. In our study, we did not examine associations with allergic rhinitis because it was not collected in the ELFE study before age of 5.5 years, and 5 years of age is too early to properly examine allergic rhinitis [40]. In the EDEN mother-child cohort, the MeDALL consortium definition [4] was used to identify children with eczema, while a simple declaration of eczema symptoms was used in the ELFE study. This could have led to an overestimation of eczema in our study. However, we have higher statistical power in the ELFE study compared to EDEN mother-child cohort which can also explain the more numerous associations observed in the present study.

Prenatal dietary exposure to a mixture of pesticides (Pest-1) was positively associated with the risk of food allergy. This association was weak but consistent across sensitivity analyses. In the individual chemicals analysis, we reported positive associations with the trace element strontium and negative associations with pesticide carbaryl and 3 mycotoxins. There are very few studies that explored effects of prenatal exposure to pesticides on food allergy. In the French PELAGIE cohort, no association was found between prenatal exposure to organophosphate pesticides and food allergy in children [49], but the sample size was small (*n* = 256) and food allergy was assessed only up to 2 y. In the French EDEN mother-child cohort, prenatal exposure to heavy metals was related to a higher risk of food allergy [50].

Another mixture composed mainly by pesticides was positively associated with the risk of wheezing. Similar findings were reported in individual chemicals analyses, even though they did not reach significance after multiple testing correction. Moreover, the association was not significant in weighted analyses nor in those conducted after exclusion of wheezing cases reported only once during the follow-up. In the CHAMACOS birth cohort an association was reported between prenatal metabolites of organophosphate pesticides concentrations and respiratory symptoms up to 7 y [51]. In our study, the main contributors to the mixture Mixt4 did not belong to organophosphates.

A negative association was highlighted between prenatal dietary exposure to a mixture composed mainly by PFAAs, PAHs and trace elements (Mixt-3) and the risk of asthma. This association was consistent across sensitivity analyses. Individual analyses also showed negative associations with several PAHs and pesticides. To our knowledge, no other study reported similar negative associations between these chemicals and asthma. Moreover, our results are contradictory with previous studies reporting higher risk of wheezing or asthma in children with higher prenatal biomarkers of exposure to PAHs [27, 31]. However, these studies used metabolites of PAHs as overall biomarkers of exposure to this group of chemicals, which does not allow to distinguish the effect of each substance. In contrast, in our study we examined exposure to 20 PAHs separately and most of the PAHs found to be negatively associated with asthma are considered as good indicators of dietary exposure to PAHs [65]. Moreover, biomarkers reflect exposure to aggregated sources of exposure such as diet and air pollution. In the atmospheric fine particulate matter (PM), some PAHs are part of multiple components that are potentially involved in asthma initiation, exacerbation and progression. Thus, it is difficult to distinguish the role of each single factor in the observed associations [30]. In the Elfe study, exposure to some pesticides that were negatively associated with asthma was highly correlated with maternal intake of fruit (r > 0.9). Then, negative associations could be explained by the potential beneficial effect of high intake of fruit and vegetable on asthma, as reported in general population [26]. However, the protective effect of fruit consumption during pregnancy on asthma and respiratory events in offspring is not well established [17]. Moreover, in our study the mixture composed by perfluoroalkyl acids, PAHs and trace elements was not strongly correlated with any individual food item. Another explanation to the negative association with this mixture observed could be epigenetic modifications through DNA hypermethylation induced by chemicals such as cadmium [54] or PFOS [37]. In fact, a DNA hypomethylation was associated with childhood asthma [66].

Several mechanisms may operate in early life to explain the associations between prenatal exposure to environmental chemicals and the risk of developing allergies later in life. Alteration of the innate immune system development, associated with lymphocyte activation (increased B-cell and IgG production) and decreased pro-inflammatory cytokines response (TNFα, IL-6, IL-33) in neonates prenatally exposed to chemicals has been previously reported [20]. Also, epigenetic regulation mechanisms such as DNA methylation were associated with prenatal exposure to some chemicals and involved in the etiology of asthma [61]. Finally, exposure to environmental factors may result in an alteration of epithelial barriers of the skin and mucosal surfaces, that have been linked to increased risk of allergies [14]. Inversely, negative associations may be explained by an immunosuppressive effect of some chemicals [59].

The ELFE study is the first nationwide birth cohort in France. The large study sample size, the prospective design, and the availability of a large set of sociodemographic data allowed adjustment on mostly all confounding factors of interest in this study. To deal with selection and attrition bias, a specific weighting was calculated. Overall, similar findings were found in the weighted analyses and the main analyses, suggesting that this bias did not have a major impact on our results. Allergic and respiratory events were reported by parents, which may lead to an overestimation of the outcomes [23]. To limit this bias, most of the used items were derived from validated questionnaires and the prospective collection of allergic or respiratory related events limited potential memory bias. Moreover, sensitivity analyses excluding individuals with only one report of the health outcomes showed robust results, except for the association between exposure to pesticides (Mixt-4) and the risk of wheezing.

Estimation of chemical exposure through diet may also suffer from a lack of precision leading to measurement errors [57, 62]. This estimation relied on combination of food intake during pregnancy with chemicals concentration in food items without integrating the placental transfer characteristics for these chemicals. Then, chemicals concentration in food items were combined with maternal dietary intake collected 5 years after chemicals assessment in food item, which might have led to additional uncertainty in the exposure estimation. Also, some chemicals and, even, some mixture of chemicals were highly correlated with a particular food item, which can lead to a confusion with the beneficial effect of this food. In particular, the PCB-BFR-Aso-MeHg mixture was strongly related to fish intake (*r* = 0.89), Pest-1 mixture to apple/pear intake (*r* = 0.97), Pest-3 mixture to grape intake (*r* = 0.92), PFAA-Ge-Li mixture to tap water (*r* = 0.97), Pest-2 mixture to strawberry/raspberry intake (*r* = 0,85) and Mixt-4 mixture to raw vegetables (*r* = 0.92). Further studies are then needed to distinguish between the potential influence of food components from those related to chemicals. However, our main findings concerned the mixtures TE-F-PAH and Mixt-3 which were not strongly correlated to any food item, suggesting that the associations are more likely to be due to the chemical content that to other food components.

Nevertheless, diet is a major source of exposure to multiple environmental chemicals, yet very few studies explored the effect of early life exposure to food chemicals on individual’s health. Most epidemiological studies exploring these associations used biomarkers of exposures, which result from multiple routes of exposure. Further, for most chemicals, biomarkers of exposure analyzed at a single time point reflect a relatively short-term exposure and depend strongly on the half-life of the considered substance, but also on endogenous metabolism that shows high inter and intra-individual variations [48]. Thus, in this study, we were able to explore the specific effect of potential dietary exposure to mixtures of chemicals, over the third trimester of pregnancy on allergic and respiratory events. Nevertheless, atmospheric, domestic, or personal care products use are also important sources of exposure to environmental chemicals and can be explored with the use of human biomonitoring data as proposed in the Helix [60] and the Athlete [3] projects. As the mixtures were data-driven, it is difficult to compare our results with previous literature. However, findings from mixtures analyses were consistent with those observed in single exposure models, as the main contributive chemicals to the mixtures significantly related to allergic outcomes are also individually associated to the same outcome.

Finally, in our study, we did not consider the potential effect of postnatal exposure to food chemicals on the studied outcomes. Considering the role of environmental exposures in the maturation of the immune system of the newborn [18], it would be interesting to investigate separately the effects of each of the pre and postnatal exposures to chemicals on allergic and respiratory diseases later in life.

## Conclusion

In our study, considering prenatal dietary exposure, only few individual chemicals showed associations with allergic diseases up to 5 years. Higher dietary exposure to a mixture of PAHs, furans and trace elements was associated with a slightly higher risk of eczema and higher exposure to mixtures of pesticides was associated with a slightly higher risk of food allergy or wheezing up to 5.5 years. Conversely, higher dietary exposure to mixture of perfluoroalkyl acids, PAHs and trace elements was associated with a lower risk of asthma. These findings highlight the importance of identifying multiple exposure patterns when investigating the potential health effects resulting in early life exposure to environmental chemicals.

### Supplementary Information

**Additional file 1: Supplementary table 1.** Food chemicals description. **Supplementary figure 1.** Directed acyclic graphs for covariate selection. **Supplementary table 2.** Multiple imputation details. **Supplementary table 3.** Associations between prenatal dietary exposure to chemicals mixtures and the risk of eczema or wheezing in childhood (exclusion of children with only 1 parental reports of the outcome). **Supplementary figure 2.** Associations between prenatal dietary exposure to individual chemicals and eczema up to 5.5 y (*n*=11,636). **Supplementary table 4.** Associations between prenatal dietary exposure to individual chemicals and eczema up to 5.5 y (with multiple testing correction). **Supplementary figure 3.** Associations between prenatal dietary exposure to individual chemicals and the risk of food allergies up to 5.5 y (*n*=11,635). **Supplementary table 5.** Associations between prenatal dietary exposure to individual chemicals and the risk of food allergy up to 5.5 y (with multiple testing correction). **Supplementary figure 4.** Associations between prenatal dietary exposure to individual chemicals and the risk of wheezing up to 5.5 y (*n*=11,636). **Supplementary table 6.** Associations between prenatal dietary exposure to individual chemicals and the risk of wheezing up to 5.5 y (with multiple testing correction). **Supplementary figure 5.** Associations between prenatal dietary exposure to individual chemicals and the risk of asthma up to 5.5 y (*n*=10,136). **Supplementary table 7.** Associations between prenatal dietary exposure to individual chemicals and the risk of asthma up to 5.5 y (with multiple testing correction)

## Data Availability

The data analyzed in this study is subject to the following licenses/restrictions. The data underlying the findings cannot be made freely available for ethical and legal restrictions imposed because this study includes a substantial number of variables that together could be used to re-identify the participants based on a few key characteristics and then used to access other personal data. Therefore, the French ethics authority strictly forbids making these data freely available. However, they can be obtained upon request from the ELFE principal investigator. Readers may contact marie-aline.charles@inserm.fr to request the data. The code book and analytic code will be made available upon request pending application and approval. Requests to access these datasets should be directed to marie-aline.charles@inserm.fr.

## Abbreviations

PFAAs: Perfluoroalkyl acids
TE: Trace elements
PAHs: Polycyclic aromatic hydrocarbons
BPA: Bisphenol A
BFRs: Brominated flame retardants
PCBs: Polychlorobiphenyls
PCA: Principal components analysis
PCs: Principal components
BKMR: Bayesian kernel machine regression
SNMU: Sparse non-negative matrix underapproximation
ELFE: Etude longitudinale Française depuis l’enfance
FFQ: Food frequency questionnaire
TDS: Total diet study
LB: Lower bound
OR: Odds ratio
SD: Standard deviation
DAG: Directed acyclic graphs
PM: Particulate matter

## References

[1] Ahn K (2014). The role of air pollutants in atopic dermatitis. J Allergy Clin Immunol.

[2] Ait Bamai Y, Goudarzi H, Araki A, Okada E, Kashino I, Miyashita C, Kishi R (2020). Effect of prenatal exposure to per- and polyfluoroalkyl substances on childhood allergies and common infectious diseases in children up to age 7 years: the Hokkaido study on environment and children's health. Environ Int.

[3] Amine I, Guillien A, Philippat C, Anguita-Ruiz A, Casas M, de Castro M, Dedele A, Garcia-Aymerich J, Granum B, Grazuleviciene R, Heude B, Haug LS, Julvez J, López-Vicente M, Maitre L, McEachan R, Nieuwenhuijsen M, Stratakis N, Vafeiadi M, Wright J, Yang T, Yuan WL, Basagaña X, Slama R, Vrijheid M, Siroux V (2023). Environmental exposures in early-life and general health in childhood. Environ Health.

[4] Anto JM, Bousquet J, Akdis M, Auffray C, Keil T, Momas I, Postma DS, Valenta R, Wickman M, Cambon-Thomsen A, Haahtela T, Lambrecht BN, Lodrup Carlsen KC, Koppelman GH, Sunyer J, Zuberbier T, Annesi-Maesano I, Arno A, Bindslev-Jensen C, De Carlo G, Forastiere F, Heinrich J, Kowalski ML, Maier D, Melen E, Smit HA, Standl M, Wright J, Asarnoj A, Benet M, Ballardini N, Garcia-Aymerich J, Gehring U, Guerra S, Hohmann C, Kull I, Lupinek C, Pinart M, Skrindo I, Westman M, Smagghe D, Akdis C, Andersson N, Bachert C, Ballereau S, Ballester F, Basagana X, Bedbrook A, Bergstrom A, von Berg A, Brunekreef B, Burte E, Carlsen KH, Chatzi L, Coquet JM, Curin M, Demoly P, Eller E, Fantini MP, von Hertzen L, Hovland V, Jacquemin B, Just J, Keller T, Kiss R, Kogevinas M, Koletzko S, Lau S, Lehmann I, Lemonnier N, Makela M, Mestres J, Mowinckel P, Nadif R, Nawijn MC, Pellet J, Pin I, Porta D, Ranciere F, Rial-Sebbag E, Saeys Y, Schuijs MJ, Siroux V, Tischer CG, Torrent M, Varraso R, Wenzel K, Xu CJ (2017). Mechanisms of the development of allergy (MeDALL): introducing novel concepts in allergy phenotypes. J Allergy Clin Immunol.

[5] Asher MI, Keil U, Anderson HR, Beasley R, Crane J, Martinez F, Mitchell EA, Pearce N, Sibbald B, Stewart AW (1995). International study of asthma and allergies in childhood (ISAAC): rationale and methods. Eur Respir J.

[6] Béchaux C, Zetlaoui M, Tressou J, Leblanc J-C, Héraud F, Crépet A (2013). Identification of pesticide mixtures and connection between combined exposure and diet. Food Chem Toxicol.

[7] Beck IH, Timmermann CAG, Nielsen F, Schoeters G, Jøhnk C, Kyhl HB, Høst A, Jensen TK (2019). Association between prenatal exposure to perfluoroalkyl substances and asthma in 5-year-old children in the Odense child cohort. Environmen Health.

[8] Berger K, Coker E, Rauch S, Eskenazi B, Balmes J, Kogut K, Holland N, Calafat AM, Harley K (2020). Prenatal phthalate, paraben, and phenol exposure and childhood allergic and respiratory outcomes: evaluating exposure to chemical mixtures. Sci Total Environ.

[9] Berlin M, Flor-Hirsch H, Kohn E, Brik A, Keidar R, Livne A, et al. Maternal exposure to polychlorinated biphenyls and asthma, allergic rhinitis and atopic dermatitis in the offspring: the environmental health fund birth cohort. Front Pharmacol. 2022;13:802974. 10.3389/fphar.2022.802974 PMC9019472 35462915

[10] Bianchi CM, Mariotti F, Verger EO, Huneau JF (2016). Pregnancy requires major changes in the quality of the diet for nutritional adequacy: simulations in the French and the United States populations. PLoS One.

[11] Billionnet C, Sherrill D, Annesi-Maesano I (2012). Estimating the health effects of exposure to multi-pollutant mixture. Ann Epidemiol.

[12] Blondel B, Lelong N, Kermarrec M, Goffinet F (2012). Trends in perinatal health in France from 1995 to 2010. Results from the French National Perinatal Surveys. J Gynecol Obstet Biol Reprod (Paris).

[13] Buralli RJ, Dultra AF, Ribeiro H (2020). Respiratory and allergic effects in children exposed to pesticides—a. Syst Rev.

[14] Celebi Sozener Z, Ozdel Ozturk B, Cerci P, Turk M, Gorgulu Akin B, Akdis M, Altiner S, Ozbey U, Ogulur I, Mitamura Y, Yilmaz I, Nadeau K, Ozdemir C, Mungan D, Akdis CA (2022). Epithelial barrier hypothesis: effect of the external exposome on the microbiome and epithelial barriers in allergic disease. Allergy.

[15] Chan-Hon-Tong A, Charles M-A, Forhan A, Heude B, Sirot V (2013). Exposure to food contaminants during pregnancy. Sci Total Environ.

[16] Charles MA, Thierry X, Lanoe J-L, Bois C, Dufourg M-N, Popa R, Cheminat M, Zaros C, Geay B (2020). Cohort profile: the French national cohort of children (ELFE): birth to 5 years. Int J Epidemiol.

[17] Delvert R, Ghozal M, Adel-Patient K, Kadawathagedara M, Heude B, Charles M-A, Annesi-Maesano I, Tafflet M, Leynaert B, Varraso R, de Lauzon-Guillain B, Bédard A (2023). Maternal diet quality during pregnancy and allergic and respiratory multimorbidity clusters in children from the EDEN mother&child cohort. Nutrients.

[18] Dietert RR, Zelikoff JT (2008). Early-life environment, developmental immunotoxicology, and the risk of pediatric allergic disease including asthma. Birth Defects Res B Dev.

[19] Donohue KM, Miller RL, Perzanowski MS, Just AC, Hoepner LA, Arunajadai S, Canfield S, Resnick D, Calafat AM, Perera FP, Whyatt RM (2013). Prenatal and postnatal bisphenol a exposure and asthma development among inner-city children. J Allergy Clin Immunol.

[20] García-Serna AM, Martín-Orozco E, Hernández-Caselles T, Morales E. Prenatal and perinatal environmental influences shaping the neonatal immune system: a focus on asthma and allergy origins. Int J Environ Res Public Health. 2021;18(8):3962. 10.3390/ijerph18083962 PMC8069583 33918723

[21] Ghozal M, Kadawathagedara M, Delvert R, Adel-Patient K, Tafflet M, Annesi-Maesano I, Crépet A, Sirot V, Charles MA, Heude B, de Lauzon-Guillain B (2023). Prenatal dietary exposure to chemicals and allergy or respiratory diseases in children in the EDEN mother-child cohort. Environ Int.

[22] Govarts E, Gilles L, Rodriguez Martin L, Santonen T, Apel P, Alvito P, Anastasi E, Andersen HR, Andersson A-M, Andryskova L, Antignac J-P, Appenzeller B, Barbone F, Barnett-Itzhaki Z, Barouki R, Berman T, Bil W, Borges T, Buekers J, Cañas-Portilla A, Covaci A, Csako Z, Den Hond E, Dvorakova D, Fabelova L, Fletcher T, Frederiksen H, Gabriel C, Ganzleben C, Göen T, Halldorsson TI, Haug LS, Horvat M, Huuskonen P, Imboden M, Jagodic Hudobivnik M, Janasik B, Janev Holcer N, Karakitsios S, Katsonouri A, Klanova J, Kokaraki V, Kold Jensen T, Koponen J, Laeremans M, Laguzzi F, Lange R, Lemke N, Lignell S, Lindroos AK, Lobo Vicente J, Luijten M, Makris KC, Mazej D, Melymuk L, Meslin M, Mol H, Montazeri P, Murawski A, Namorado S, Niemann L, Nübler S, Nunes B, Olafsdottir K, Palkovicova Murinova L, Papaioannou N, Pedraza-Diaz S, Piler P, Plichta V, Poteser M, Probst-Hensch N, Rambaud L, Rauscher-Gabernig E, Rausova K, Remy S, Riou M, Rosolen V, Rousselle C, Rüther M, Sarigiannis D, Silva MJ, Šlejkovec Z, Snoj Tratnik J, Stajnko A, Szigeti T, Tarazona JV, Thomsen C, Tkalec Ž, Tolonen H, Trnovec T, Uhl M, Van Nieuwenhuyse A, Vasco E, Verheyen VJ, Viegas S, Vinggaard AM, Vogel N, Vorkamp K, Wasowicz W, Weber T, Wimmerova S, Woutersen M, Zimmermann P, Zvonar M, Koch H, Kolossa-Gehring M, Esteban López M, Castaño A, Stewart L, Sepai O, Schoeters G (2023). Harmonized human biomonitoring in European children, teenagers and adults: EU-wide exposure data of 11 chemical substance groups from the HBM4EU aligned studies (2014–2021). Int J Hyg Environ Health.

[23] Grabenhenrich L, Trendelenburg V, Bellach J, Yürek S, Reich A, Fiandor A, Rivero D, Sigurdardottir S, Clausen M, Papadopoulos NG, Xepapadaki P, Sprikkelman AB, Dontje B, Roberts G, Grimshaw K, Kowalski ML, Kurowski M, Dubakiene R, Rudzeviciene O, Fernández-Rivas M, Couch P, Versteeg SA, van Ree R, Mills C, Keil T, Beyer K (2020). Frequency of food allergy in school-aged children in eight European countries-the EuroPrevall-iFAAM birth cohort. Allergy.

[24] Grados L, Pérot M, Barbezier N, Delayre-Orthez C, Bach V, Fumery M, et al. How advanced are we on the consequences of oral exposure to food contaminants on the occurrence of chronic non communicable diseases? Chemosphere. 2022;303(Part 3):135260. 10.1016/j.chemosphere.2022.135260 35688194

[25] Granum B, Oftedal B, Agier L, Siroux V, Bird P, Casas M, Warembourg C, Wright J, Chatzi L, de Castro M, Donaire D, Grazuleviciene R, Småstuen Haug L, Maitre L, Robinson O, Tamayo-Uria I, Urquiza J, Nieuwenhuijsen M, Slama R, Thomsen C, Vrijheid M (2020). Multiple environmental exposures in early-life and allergy-related outcomes in childhood. Environ Int.

[26] Hosseini B, Berthon BS, Wark P, Wood LG (2017). Effects of fruit and vegetable consumption on risk of asthma, wheezing and immune responses: a systematic review and Meta-analysis. Nutrients.

[27] Jedrychowski WA, Perera FP, Maugeri U, Mrozek-Budzyn D, Mroz E, Klimaszewska-Rembiasz M, Flak E, Edwards S, Spengler J, Jacek R, Sowa A (2010). Intrauterine exposure to polycyclic aromatic hydrocarbons, fine particulate matter and early wheeze. Prospective birth cohort study in 4-year olds. Pediatr Allergy Immunol.

[28] Juillard, H. (2015). "Weighting of Elfe survey data at time 0. (2015) pandora.vjf.inserm.fr/public/.".

[29] Kadawathagedara M, Ahluwalia N, Dufourg M-N, Forhan A, Charles MA, Lioret S, de Lauzon-Guillain B (2021). Diet during pregnancy: influence of social characteristics and migration in the ELFE cohort. Matern Child Nutr.

[30] Karimi P, Peters KO, Bidad K, Strickland PT (2015). Polycyclic aromatic hydrocarbons and childhood asthma. Eur J Epidemiol.

[31] Kim S, Carson KA, Chien AL (2022). The association between urinary polycyclic aromatic hydrocarbon metabolites and atopic triad by age and body weight in the US population. J Dermatol Treat.

[32] Kim JH, Jeong KS, Ha EH, Park H, Ha M, Hong YC, Lee SJ, Lee KY, Jeong J, Kim Y. Association between prenatal exposure to cadmium and atopic dermatitis in infancy. J Korean Med Sci. 2013;28(4):516-21. 10.3346/jkms.2013.28.4.516. 10.3346/jkms.2013.28.4.516 PMC3617302 23580123

[33] Kim J, Kim S, Woo SY, Chung JY, Hong YS, Oh SY, Choi SJ, Oh SY, Kim KW, Shin YH, Won HS, Lee KJ, Kim SH, Kwon JY, Lee SH, Hong SJ, Ahn K. Prenatal Exposure to Lead and Chromium is Associated with IL-13 Levels in Umbilical Cord Blood and Severity of Atopic Dermatitis: COCOA Study. Immune Netw. 2019;19(6):e42. 10.4110/in.2019.19.e42. 10.4110/in.2019.19.e42 PMC6943175 31921472

[34] Koh TK, Park H, Hong YC, Ha M, Kim Y, Lee BE, Shah S, Ha E (2021). Association between prenatal polycyclic aromatic hydrocarbons and infantile allergic diseases modified by maternal glutathione S-transferase polymorphisms: results from the MOCEH birth cohort. Ann Occup Environ Med.

[35] Koh HY, Kim TH, Sheen YH, Lee SW, An J, Kim MA, Han MY, Yon DK. Serum heavy metal levels are associated with asthma, allergic rhinitis, atopic dermatitis, allergic multimorbidity, and airflow obstruction. J Allergy Clin Immunol Pract. 2019;7(8):2912-5.e2. 10.1016/j.jaip.2019.05.015. 10.1016/j.jaip.2019.05.015 31129074

[36] Lee S, Park SK, Park H, Lee W, Lee JH, Hong Y-C, et al. Joint association of prenatal bisphenol-a and phthalates exposure with risk of atopic dermatitis in 6-month-old infants. Sci Total Environ. 2021;789:147953. 10.1016/j.scitotenv.2021.147953 34323845

[37] Liu Y, Eliot MN, Papandonatos GD, Kelsey KT, Fore R, Langevin S, Buckley J, Chen A, Lanphear BP, Cecil KM, Yolton K, Hivert M-F, Sagiv SK, Baccarelli AA, Oken E, Braun JM (2022). Gestational Perfluoroalkyl substance exposure and DNA methylation at birth and 12 years of age: a longitudinal Epigenome-wide association study. Environ Health Perspect.

[38] Luijten M, Vlaanderen J, Kortenkamp A, Antignac J-P, Barouki R, Bil W, van den Brand A, den Braver-Sewradj S, van Klaveren J, Mengelers M, Ottenbros I, Rantakokko P, Kolossa-Gehring M, Lebret E (2023). Mixture risk assessment and human biomonitoring: lessons learnt from HBM4EU. Int J Hyg Environ Health.

[39] Mason CH, Perreault WD (1991). Collinearity, power, and interpretation of multiple regression analysis. J Mark Res.

[40] Meltzer EO, Blaiss MS, Derebery MJ, Mahr TA, Gordon BR, Sheth KK, Simmons AL, Wingertzahn MA, Boyle JM (2009). Burden of allergic rhinitis: results from the pediatric allergies in America survey. J Allergy Clin Immunol.

[41] Mora, A. M., J. A. Hoppin, L. Córdoba, J. C. Cano, M. Soto-Martínez, B. Eskenazi, C. H. Lindh and B. van Wendel de Joode (2020). "Prenatal pesticide exposure and respiratory health outcomes in the first year of life: results from the infants’ environmental health (ISA) study." Int J Hyg Environ Health 225: 113474. 10.1016/j.ijheh.2020.113474 PMC7058516 32066110

[42] Mori C, Igoshi Y, Ochiai S, Suzuki S, Matsuno Y, Todaka E, et al. Fetal exposure to polybrominated diphenyl ethers and atopic dermatitis of infants. Toxicology Letters. 2012;211:S94.

[43] Ochiai S, Shimojo N, Yuka I, Watanabe M, Matsuno Y, Suzuki S, Kohno Y, Mori C (2014). A pilot study for foetal exposure to multiple persistent organic pollutants and the development of infant atopic dermatitis in modern Japanese society. Chemosphere.

[44] Okada E, Sasaki S, Kashino I, Matsuura H, Miyashita C, Kobayashi S, Itoh K, Ikeno T, Tamakoshi A, Kishi R (2014). Prenatal exposure to perfluoroalkyl acids and allergic diseases in early childhood. Environ Int.

[45] Papadopoulou E, Haug LS, Sakhi AK, Andrusaityte S, Basagana X, Brantsaeter AL, Casas M, Fernandez-Barres S, Grazuleviciene R, Knutsen HK, Maitre L, Meltzer HM, McEachan RRC, Roumeliotaki T, Slama R, Vafeiadi M, Wright J, Vrijheid M, Thomsen C, Chatzi L (2019). Diet as a source of exposure to environmental contaminants for pregnant women and children from six European countries. Environ Health Perspect.

[46] Parker-Lalomio M, McCann K, Piorkowski J, Freels S, Persky VW (2018). Prenatal exposure to polychlorinated biphenyls and asthma, eczema/hay fever, and frequent ear infections. J Asthma.

[47] Pat Y, Ogulur I, Yazici D, Mitamura Y, Cevhertas L, Küçükkase OC, Mesisser SS, Akdis M, Nadeau K, Akdis CA (2023). Effect of altered human exposome on the skin and mucosal epithelial barrier integrity. Tissue Barriers.

[48] Paustenbach D, Galbraith D (2006). Biomonitoring and biomarkers: exposure assessment will never be the same. Environ Health Perspect.

[49] Pelé, F., Q. Vieille, F. Rouget, C. Monfort, S. Cordier and C. Chevrier (2016). "Prenatal exposure to Organophosphate Pesticides and infant respiratory and allergic outcomes in the PELAGIE cohort." ISEE Conference Abstracts 2016.

[50] Pesce G, Sesé L, Calciano L, Travert B, Dessimond B, Maesano CN, Ferrante G, Huel G, Prud’homme J, Guinot M, Soomro MH, Baloch RM, Lhote R, Annesi-Maesano I (2021). Foetal exposure to heavy metals and risk of atopic diseases in early childhood. Pediatr Allergy Immunol.

[51] Raanan R, Harley KG, Balmes JR, Bradman A, Lipsett M, Eskenazi B (2015). Early-life Exposure to Organophosphate Pesticides and Pediatric Respiratory Symptoms in the CHAMACOS Cohort. Environ Health Perspect.

[52] Rager JE, Bangma J, Carberry C, Chao A, Grossman J, Lu K, Manuck TA, Sobus JR, Szilagyi J, Fry RC (2020). Review of the environmental prenatal exposome and its relationship to maternal and fetal health. Reprod Toxicol.

[53] Ruan F, Zhang J, Liu J, Sun X, Li Y, Xu S, Xia W (2022). Association between prenatal exposure to metal mixtures and early childhood allergic diseases. Environ Res.

[54] Ruiz-Hernandez A, Kuo C-C, Rentero-Garrido P, Tang W-Y, Redon J, Ordovas JM, Navas-Acien A, Tellez-Plaza M (2015). Environmental chemicals and DNA methylation in adults: a systematic review of the epidemiologic evidence. Clin Epigenetics.

[55] Sandys O, Te Velde A (2022). Raising the alarm: environmental factors in the onset and maintenance of chronic (low-grade) inflammation in the gastrointestinal tract. Dig Dis Sci.

[56] Shrier I, Platt RW (2008). Reducing bias through directed acyclic graphs. BMC Med Res Methodol.

[57] Sirot V, Volatier JL, Calamassi-Tran G, Dubuisson C, Menard C, Dufour A, Leblanc JC (2009). Core food of the French food supply: second Total diet study. Food Addit Contam Part A Chem Anal Control Expo Risk Assess.

[58] Smit LAM, Lenters V, Høyer BB, Lindh CH, Pedersen HS, Liermontova I, Jönsson BAG, Piersma AH, Bonde JP, Toft G, Vermeulen R, Heederik D (2015). Prenatal exposure to environmental chemical contaminants and asthma and eczema in school-age children. Allergy.

[59] Sun Y, Huang K, Long M, Yang S, Zhang Y (2022). An update on immunotoxicity and mechanisms of action of six environmental mycotoxins. Food Chem Toxicol.

[60] Tamayo-Uria I, Maitre L, Thomsen C, Nieuwenhuijsen MJ, Chatzi L, Siroux V, Aasvang GM, Agier L, Andrusaityte S, Casas M, de Castro M, Dedele A, Haug LS, Heude B, Grazuleviciene R, Gutzkow KB, Krog NH, Mason D, McEachan RRC, Meltzer HM, Petraviciene I, Robinson O, Roumeliotaki T, Sakhi AK, Urquiza J, Vafeiadi M, Waiblinger D, Warembourg C, Wright J, Slama R, Vrijheid M, Basagaña X (2019). The early-life exposome: description and patterns in six European countries. Environ Int.

[61] Thürmann L, Klös M, Mackowiak SD, Bieg M, Bauer T, Ishaque N, Messingschlager M, Herrmann C, Röder S, Bauer M, Schäuble S, Faessler E, Hahn U, Weichenhan D, Mücke O, Plass C, Borte M, von Mutius E, Stangl GI, Lauener R, Karvonen AM, Divaret-Chauveau A, Riedler J, Heinrich J, Standl M, von Berg A, Schaaf B, Herberth G, Kabesch M, Eils R, Trump S, Lehmann I (2023). Global hypomethylation in childhood asthma identified by genome-wide DNA-methylation sequencing preferentially affects enhancer regions. Allergy.

[62] Traore T, Forhan A, Sirot V, Kadawathagedara M, Heude B, Hulin M, de Lauzon-Guillain B, Botton J, Charles MA, Crepet A (2018). To which mixtures are French pregnant women mainly exposed? A combination of the second French total diet study with the EDEN and ELFE cohort studies. Food Chem Toxicol.

[63] Tsatsakis AM, Katsikantami I, Kalantzi O-I, Sevim Ç, Tsarouhas K, Sarigiannis D, Tzatzarakis MN, Rizos AK (2019). Phthalates: exposure and health effects. Encyclopedia of Environmental Health (Second Edition) J Nriagu Oxford.

[64] Wang J, Yin J, Hong X, Liu R. Exposure to Heavy Metals and Allergic Outcomes in Children: a Systematic Review and Meta-analysis. Biol Trace Elem Res. 2022;200(11):4615-31. 10.1007/s12011-021-03070-w. 10.1007/s12011-021-03070-w 35006554

[65] Veyrand B, Sirot V, Durand S, Pollono C, Marchand P, Dervilly-Pinel G, Tard A, Leblanc J-C, Le Bizec B (2013). Human dietary exposure to polycyclic aromatic hydrocarbons: results of the second French Total diet study. Environ Int.

[66] Xu C-J, Söderhäll C, Bustamante M, Baïz N, Gruzieva O, Gehring U, Mason D, Chatzi L, Basterrechea M, Llop S, Torrent M, Forastiere F, Fantini MP, Carlsen KCL, Haahtela T, Morin A, Kerkhof M, Merid SK, van Rijkom B, Jankipersadsing SA, Bonder MJ, Ballereau S, Vermeulen CJ, Aguirre-Gamboa R, de Jongste JC, Smit HA, Kumar A, Pershagen G, Guerra S, Garcia-Aymerich J, Greco D, Reinius L, McEachan RRC, Azad R, Hovland V, Mowinckel P, Alenius H, Fyhrquist N, Lemonnier N, Pellet J, Auffray C, van der Vlies P, van Diemen CC, Li Y, Wijmenga C, Netea MG, Moffatt MF, Cookson WOCM, Anto JM, Bousquet J, Laatikainen T, Laprise C, Carlsen K-H, Gori D, Porta D, Iñiguez C, Bilbao JR, Kogevinas M, Wright J, Brunekreef B, Kere J, Nawijn MC, Annesi-Maesano I, Sunyer J, Melén E, Koppelman GH (2018). DNA methylation in childhood asthma: an epigenome-wide meta-analysis. Lancet Respir Med.

[67] Zetlaoui M, Feinberg M, Verger P, Clémençon S (2011). Extraction of food consumption systems by nonnegative matrix factorization (NMF) for the assessment of food choices. Biometrics.

[68] Zhou A, Chang H, Huo W, Zhang B, Hu J, Xia W, Chen Z, Xiong C, Zhang Y, Wang Y, Xu S, Li Y (2017). Prenatal exposure to bisphenol a and risk of allergic diseases in early life. Pediatr Res.

