# Supplementary material for: Prenatal dietary exposure to mixtures of chemicals is associated with allergy or respiratory diseases in children in the ELFE nationwide cohort
Source: Environ Health. 2024 Jan 9;23:5. doi: 10.1186/s12940-023-01046-y (PMC10775451; doi:10.1186/s12940-023-01046-y)
Supplement: Supplementary file 1 — Additional file 1: Supplementary table 1. Food chemicals description. Supplementary figure 1. Directed acyclic graphs for covariate selection. Supplementary table 2. Multiple imputation details. Supplementary table 3. Associations between prenatal dietary exposure to chemicals mixtures and the risk of eczema or wheezing in childhood (exclusion of children with only 1 parental reports of the outcome). Supplementary figure 2. Associations between prenatal dietary exposure to individual chemicals and eczema up to 5.5 y (n=11,636). Supplementary table 4. Associations between prenatal dietary exposure to individual chemicals and eczema up to 5.5 y (with multiple testing correction). Supplementary figure 3. Associations between prenatal dietary exposure to individual chemicals and the risk of food allergies up to 5.5 y (n=11,635). Supplementary table 5. Associations between prenatal dietary exposure to individual chemicals and the risk of food allergy up to 5.5 y (with multiple testing correction). Supplementary figure 4. Associations between prenatal dietary exposure to individual chemicals and the risk of wheezing up to 5.5 y (n=11,636). Supplementary table 6. Associations between prenatal dietary exposure to individual chemicals and the risk of wheezing up to 5.5 y (with multiple testing correction). Supplementary figure 5. Associations between prenatal dietary exposure to individual chemicals and the risk of asthma up to 5.5 y (n=10,136). Supplementary table 7. Associations between prenatal dietary exposure to individual chemicals and the risk of asthma up to 5.5 y (with multiple testing correction) [file 12940_2023_1046_MOESM1_ESM.docx]

- Online supplementary material –

PRENATAL DIETARY EXPOSURE TO MIXTURES OF CHEMICALS IS ASSOCIATED WITH ALLERGY OR RESPIRATORY DISEASES IN CHILDREN IN THE ELFE NATIONWIDE COHORT

**Supplementary materials caption**

Supplementary table 1. Food chemicals description.

Supplementary figure 1: Directed acyclic graphs for covariate selection

Supplementary table 2: Multiple imputation details

**Supplementary table 3:** Associations between prenatal dietary exposure to chemicals mixtures and the risk of eczema or wheezing in childhood (exclusion of children with only 1 parental reports of the outcome)

Supplementary figure 2: Associations between prenatal dietary exposure to individual chemicals and eczema up to 5.5 y (n=11,636).

Supplementary table 4: Associations between prenatal dietary exposure to individual chemicals and eczema up to 5.5 y (with multiple testing correction).

Supplementary figure 3: Associations between prenatal dietary exposure to individual chemicals and the risk of food allergies up to 5.5 y (n=11,635).

Supplementary table 5: Associations between prenatal dietary exposure to individual chemicals and the risk of food allergy up to 5.5 y (with multiple testing correction)

Supplementary figure 4: Associations between prenatal dietary exposure to individual chemicals and the risk of wheezing up to 5.5 y (n=11,636).

Supplementary table 6: Associations between prenatal dietary exposure to individual chemicals and the risk of wheezing up to 5.5 y (with multiple testing correction)

Supplementary figure 5: Associations between prenatal dietary exposure to individual chemicals and the risk of asthma up to 5.5 y (n=10,136).

Supplementary table 7: Associations between prenatal dietary exposure to individual chemicals and the risk of asthma up to 5.5 y (with multiple testing correction)

**Supplementary table 1.** Food chemicals description

| Groups | | Chemicals |
| --- | --- | --- |
| Pesticides | |  |
|  | *Organochlorine* | Hexachlorobenzene*, endosulfan*, lindane* |
|  | *Organophosphorus* | Azinphos_methyl*, chlorpyrifos_ethyl*, chlorpyrifos_methyl, diazinon*, dichlorvos*, dimethoate*, ethion*, fenitrothion*, malathion*, phosalone*, phosmet*, pirimiphos_methyl, chlorfenvinphos* |
|  | *Pyretrinoids* | Acrinathrin*, bifenthrin*, cyfluthrin*, etofenprox*, lambda_cyhalothrin*, permethrin*, piperonyl |
|  | *Carbamates* | Carbaryl*, carbofuran*, carbendazim, chlorpropham, diethofencarb*, methomyl*, pirimicarb* |
|  | *Diraboximides* | Captan*, folpet*, iprodione, procymidone*, vinclozolin* |
|  | *Benzoylureas* | Diflubenzuron*, teflubenzuron*, triflumuron* |
|  | *Triazoles* | Cyproconazole*, fenbuconazole*, flutriafol*, myclobutanil*, penconazole*, tebuconazole*, tetraconazole*, triadimenol* |
|  | *Other pesticides* | Diphenylamine*, cyprodinil, mepanipyrim*, pyrimethanil*, tebufenozide*, chlortal*, boscalid*, tetradifon*, fenhexamid*, imazalil, pyriproxyfen*, fludioxonil, tebufenpyrad*, bupirimate*, quinoxyfen*, spiroxamine*, trifloxystrobin*, azoxystrobin*, kresoxim_methyl*, propargite*, p2_phenylphenol, metalaxyl_m*, thiabendazole*, chlorothalonil*, ethoxyquin*, sulfur*, imidacloprid* |
| Trace elements | | Aluminum (Al), inorganic and organic arsenic (Asi and Aso) arsenic, barium (Ba), cadmium (Cd), cobalt (Co), trivalent and tetravalent chromium (CrIII and CrVI), copper (Cu), gallium (Ga)*, germanium (Ge), inorganic and methyl mercury (Hgi, and MeHg )*, lithium (Li), nickel (Ni), lead (Pb), antimony (Sb), tin (Sn), strontium (Sr), tellurium (Te) and vanadium (V) |
| PAHs | | Anthrancene (AN), benzo [a]anthracene (BaA), benzo[a]pyrene (BaP), benzo[b]fluoranthene (BcFL), benzo[c]fluorine (BbF), benzo[g,h,i]perylene (BghiP), benzo[j]fluoranthene (BjF), benzo[k]fluoranthene (BkF), chrysene (CHR), cyclopenta[c,d]pyrene (CPP), dibenzo[a,e]pyrene (DbaeP), dibenzo[a,h]anthracene (DBahA), dibenzo[a,h]pyrene (DbahP)*, dibenzo[a,i]pyrene (DbaiP)*, dibenzo[a,l]pyrene (DbalP)*, fluoranthene (FA), indeno [1,2,3-cd]pyrene (IP), methylchrysene (MCH)*, phenanthrene (PHE), pyrene (PY) |
| PCBs | | PCB_156, PCB_157, PCB_167, PCB_169, PCB_180, PCB_189, PCB_28, PCB_52, PCB_77, PCB_81, PCB_101, PCB_105, PCB_114, PCB_118, PCB_123, PCB_126, PCB_138, PCB_153 |
| Dioxins and furans | | Hexachlorodibenzo-p-dioxine_1234678 (HCDD_1234678), HCDD_123478, HCDD_123678, HCDD_123789, octachlorodibenzodioxine (OCDD), polychlorodibenzo-p-dioxine_12378 (PCDD_1237), tétrachlorodibenzo-p-dioxine_2378 (TCDD_2378), hexachlorodibenzo-furan_1234678 (HCDF_1234678), HCDF_1234789, HCDF_123478, HCDF_123678, HCDF_123789, HCDF_234678, octachlorodibenzofuran, polychlorodibenzofurans_12378 (PCDF_12378), PCDF_23478 and tetrachlorodibenzofurans_2337 (TCDF) |
| Mycotoxins | | Alpha-zearalanol (zea)*, alpha-zearalenol (zee)*, diacetoxyscirpenol (DAS)*, deoxynivalenol (DON), de-epoxy derivative of DON (DOM1), 15-acetyldeoxynivalenol (DON15)*, 3-acetyldeoxynivalenol (DON3)*, fumonisin-B1 (FB1), fumonisin-B2 (FB2), fusarenon-X (FusX)*, HT2-toxin (HT2), monoacetoxyscirpenol (MAS), nivalenol (Niv), ochratoxin-A (OTA), ochratoxin-B (OTB)*, Patulin (Pat), T2-toxin (T2) and zeralenone (zer) |
| BFRs | | Alpha-Hexabromocyclododecane (HBCDalpha), beta-Hexabromocyclododecane (HBCDbeta)*, gamma-Hexabromocyclododecane (HBCDgamma)*, pentabromobiphenyl 101 (PBB101)*, PBB153*, PBB52*, polybrominated diphenyl ether 100 (PBDE100)*, PBDE153*, PBDE154*, PBDE183, PBDE209, PBDE28*, PBDE47, PBDE99 |
| PFAAs | | Perfluorobutane sulfonate (PFBS)*, perfluorodecanoic acid (PFDA)*, perfluorododecanoic acid (PFDoA)*, perfluoroheptanoic acid (PFHpA)*, perfluorohexanoic acid (PFHxA)*, perfluorohexane sulfonate (PFHxS)*, perfluorononanoic acid (PFNA)*, perfluorooctanoic acid (PFOA), perfluorooctane sulfonate (PFOS), perfluorotetradecanoic acid (PFTeDA)*, perfluorotridecanoic acid (PFTrDA)* and perfluoroundecanoic acid (PFUnA)* |
| Phytoestrogens | | Biochanin_A, resveratrol*, coumestrol, daidzein, enterolactone, equol, formononetin, genistein, glycitein, matairesinol* and secoisolariciresinol |
| Additives | | Tartaric acid, nitrites, sulfites, rocou* |
| Other chemicals | | Bisphenol A (BPA), acrylamide |

* Chemicals categorised in terciles for analysis

BFRs: Brominated flame retardants; PAHs: polycyclic aromatic hydrocarbons; PFAAs: perfluoroalkyl acids; PCBs: polychlorinated biphenyls.


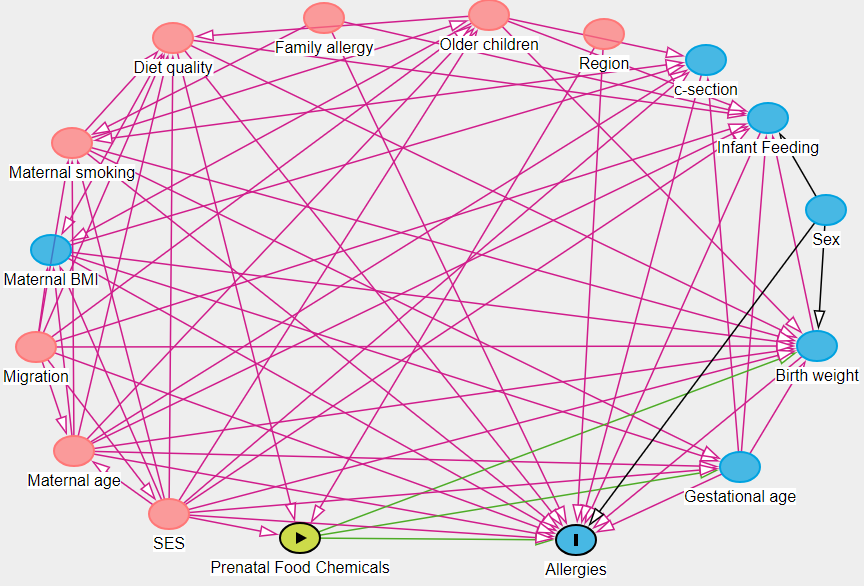


**Supplementary figure 1:** Directed acyclic graphs for covariate selection

The green circle with the triangle is the studied exposure (maternal exposure to food chemicals during pregnancy) and the blue circle with the “I” is the studied outcome (allergic or respiratory diseases in childhood). Red circles represent potential confounders and blue circles are variables not expected to influence maternal exposure to food chemicals but related to allergies. Arrows denote the direction of causal association**.**

**Supplementary table 2:** Multiple imputation details

| Variable | Variable type | Model used to predict missing data | Missing values |
| --- | --- | --- | --- |
| 112 food chemicals | Continuous | No missing data | 0 |
| 98 food chemicals | Binary | No missing data | 0 |
| Wheezing from 0 to 5.5 years | Binary | No missing data | 0 |
| Current asthma (ISAAC definition) from 0 to 5.5 years | Binary | No missing data | 0 |
| Eczema from 0 to 5.5 years | Binary | No missing data | 0 |
| Food allergy from 0 to 5.5 years | Binary | No missing data | 0 |
| Study wave | Categorical (4 categories) | No missing data | 0 |
| Maternity size | Ordinal (5 categories) | No missing data | 0 |
| Maternal diet quality | Continuous | No missing data | 0 |
| Maternal age | Continuous | Linear regression (pmm) | 1 (0.007%) |
| Child sex | Binary | Logistic regression | 1 (0.007%) |
| Maternal rural residence | Binary | Logistic regression | 2 (0.01%) |
| Maternal region of residence | Categorical (9 categories) | Multinomial regression | 3 (0.02%) |
| Mode of delivery | Binary | Logistic regression | 89 (0.7%) |
| Maternal smoking during pregnancy | Ordinal (4 categories) | Logistic regression | 120 (0.9%) |
| Older children in the household | Ordinal (3 categories) | Logistic regression | 231 (1.7%) |
| Maternal employment status during pregnancy | Binary | Logistic regression | 251 (1.9%) |
| Maternal migration status | Categorical (3 categories) | Multinomial regression | 299 (2.2%) |
| Family history of allergies | Binary | Logistic regression | 371 (2.8%) |
| Maternal education level | Ordinal (5 categories) | Logistic regression | 642 (4.8%) |
| Household income | Continuous | Linear regression (pmm) | 721 (5.4%) |

pmm: predictive mean matching

**Supplementary table 3:** Associations between prenatal dietary exposure to chemicals mixtures and the risk of eczema or wheezing in childhood (exclusion of cases with only 1 parental report of the outcome)

| Mixture name | OR [95% CI] | | | |
| --- | --- | --- | --- | --- |
|  | **Eczema**  **(n=8,208)** | **Food allergy**  **(n=11,036)** | **Wheezing**  **(n=8,551)** | **Asthma**  **(n=9,432)** |
| TE-F-PAH | 1.12 [1.06; 1.19] | 1.18 [0.95; 1.47] | 1.03 [0.96; 1.11] | 0.96 [0.76; 1.22] |
| Mixt-3 | 0.97 [0.92; 1.02] | 0.68 [0.48; 0.97] | 0.98 [0.92; 1.04] | 0.67 [0.46; 0.99] |
| Pest-1 | 0.99 [0.95; 1.04] | 1.19 [1.02; 1.39] | 0.93 [0.87; 0.99] | 0.95 [0.77; 1.17] |
| Pest-3 | 0.99 [0.95; 1.04] | 1.06 [0.90; 1.26] | 1.00 [0.94; 1.06] | 0.93 [0.75; 1.17] |
| PCB-BFR-Aso-MeHg | 0.98 [0.93; 1.03] | 0.92 [0.74; 1.15] | 0.96 [0.90; 1.03] | 0.97 [0.78; 1.22] |
| Pest-2 | 0.98 [0.93; 1.03] | 0.89 [0.73; 1.09] | 1.04 [0.97; 1.10] | 1.04 [0.88; 1.24] |
| Mixt-4 | 0.99 [0.95; 1.04] | 1.00 [0.84; 1.18] | 1.01 [0.95; 1.07] | 0.92 [0.76; 1.12] |
| PFAA-Ge-Li | 1.01 [0.96; 1.05] | 0.95 [0.79; 1.14] | 0.99 [0.93; 1.05] | 0.97 [0.80; 1.17] |

Values are odds ratios [95% CI] from logistic regression models including the eight mixtures simultaneously and adjusted for maternal characteristics (age, education level, migration status, employment, household income, rural residence, region of residence, number of older children in the household, smoking during pregnancy, diet quality), child and birth characteristics (sex, mode of delivery, family history of allergies), and variables related to study design (maternity size and recruitment wave). Abbreviations: TE-F-PAH, trace elements-furans-polycyclic aromatic hydrocarbons; PCB-BFR-Aso-MeHg, polychlorobiphenyls-brominated flame retardants-organic arsenic-methylmercury; Pest-1, pesticides-1; Pest-2, pesticides-2; Pest-3, pesticides-3; PFAA-Ge-Li, perfluoroalkyl acids-germanium-lithium; Mixt-3, mixture-3; Mixt-4, mixture-4.


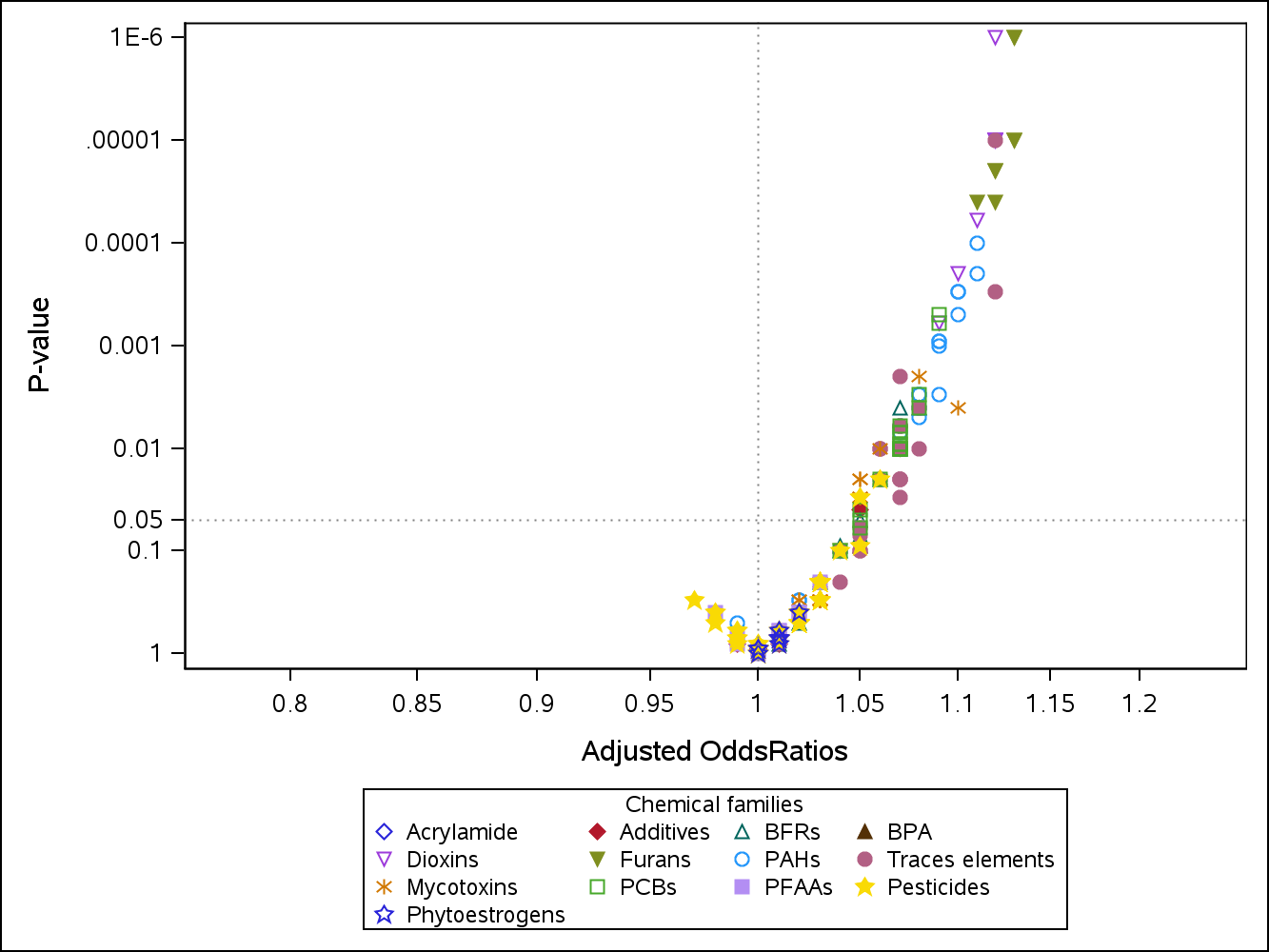


**Supplementary figure 2:** Associations between prenatal dietary exposure to individual chemicals and eczema in children up to 5.5 y (n=11,636).

Odds ratios (OR) were calculated using a logistic regression model for each chemical separately and adjusted for maternal characteristics (age, education level, migration status, employment, household income, maternal rural residence, maternal region of residence, number of older children in the household, smoking during pregnancy, diet quality), child and birth characteristics (sex, mode of delivery, family history of allergies), maternity size and study wave. Prenatal exposure to food chemicals was log-transformed and then standardized. BPA: Bisphenol A; BFRs: Brominated flame retardants; PAHs: polycyclic aromatic hydrocarbons; PFAAs: perfluoroalkyl acids; PCBs: polychlorinated biphenyls.

**Supplementary table 4:** Associations between prenatal dietary exposure to individual chemicals and eczema up to 5.5 y (with multiple testing correction)

|  | Multiple imputation  (n=11,636) | | | Complete case  (n=9,908) | | | Weighted analysis  (n=11,636) | | |
| --- | --- | --- | --- | --- | --- | --- | --- | --- | --- |
| Food chemical | **OR [95% CI]** | **p value** | **q value** | **OR [95% CI]** | **p value** | **q value** | **OR [95% CI]** | **p value** | **q value** |
| PBDE183 | 1.07 [1.01; 1.12] | 0.01 | 0.015 | 1.04 [0.99; 1.09] | 0.1 | 0.13 | 1.08 [1.02; 1.16] | 0.01 | 0.07 |
| PBDE209 | 1.06 [1.01; 1.11] | 0.02 | 0.03 | 1.04 [0.99; 1.08] | 0.1 | 0.13 | 1.06 [1.00; 1.12] | 0.07 | 0.18 |
| PBDE99 | 1.08 [1.02; 1.14] | 0.004 | 0.009 | 1.04 [1.00; 1.09] | 0.07 | 0.12 | 1.06 [1.00; 1.13] | 0.07 | 0.18 |
| HBCDbeta | 1.07 [1.02; 1.12] | 0.004 | 0.009 | 1.12 [1.03; 1.22] | 0.006 | 0.04 | 1.09 [1.03; 1.15] | 0.003 | 0.03 |
| HCDD-1234678 | 1.10 [1.05; 1.16] | 0.0002 | 0.001 | 1.07 [1.03; 1.13] | 0.002 | 0.02 | 1.09 [1.02; 1.16] | 0.02 | 0.09 |
| HCDD-123478 | 1.12 [1.07; 1.18] | 0.00001 | 0.0001 | 1.09 [1.04; 1.14] | 0.0002 | 0.01 | 1.10 [1.03; 1.17] | 0.003 | 0.03 |
| HCDD-123678 | 1.12 [1.07; 1.18] | 0.00001 | 0.0001 | 1.09 [1.04; 1.14] | 0.0003 | 0.01 | 1.11 [1.04; 1.18] | 0.002 | 0.03 |
| HCDD-123789 | 1.12 [1.07; 1.18] | <0.00001 | <0.00001 | 1.09 [1.05; 1.14] | 0.0001 | 0.01 | 1.12 [1.05; 1.19] | 0.0006 | 0.02 |
| OCDD | 1.09 [1.04; 1.15] | 0.0006 | 0.002 | 1.06 [1.02; 1.11] | 0.009 | 0.04 | 1.07 [1.00; 1.14] | 0.04 | 0.13 |
| PCDD-1237 | 1.12 [1.06; 1.17] | 0.00001 | 0.0001 | 1.09 [1.04; 1.14] | 0.0003 | 0.01 | 1.10 [1.04; 1.17] | 0.002 | 0.03 |
| TCDD-2378 | 1.11 [1.05; 1.17] | 0.00006 | 0.0004 | 1.08 [1.03; 1.13] | 0.002 | 0.02 | 1.09 [1.03; 1.17] | 0.006 | 0.05 |
| HCDF-1234678 | 1.12 [1.06; 1.18] | 0.00004 | 0.0003 | 1.08 [1.03; 1.13] | 0.002 | 0.02 | 1.11 [1.03; 1.18] | 0.003 | 0.03 |
| HCDF-123478 | 1.13 [1.07; 1.19] | 0.00001 | 0.0001 | 1.09 [1.04; 1.14] | 0.0004 | 0.01 | 1.11 [1.04; 1.19] | 0.001 | 0.02 |
| HCDF-1234789 | 1.12 [1.07; 1.19] | 0.00002 | 0.0002 | 1.08 [1.03; 1.14] | 0.0009 | 0.01 | 1.12 [1.04; 1.19] | 0.001 | 0.02 |
| HCDF-123678 | 1.13 [1.07; 1.19] | <0.00001 | <0.00001 | 1.09 [1.04; 1.14] | 0.0002 | 0.01 | 1.12 [1.05; 1.19] | 0.0008 | 0.02 |
| HCDF-123789 | 1.13 [1.08; 1.19] | <0.00001 | <0.00001 | 1.09 [1.04; 1.14] | 0.0003 | 0.01 | 1.13 [1.06; 1.20] | 0.0003 | 0.02 |
| HCDF-234678 | 1.12 [1.07; 1.19] | 0.00002 | 0.0002 | 1.08 [1.03; 1.14] | 0.001 | 0.01 | 1.11 [1.04; 1.19] | 0.002 | 0.03 |
| OCDF | 1.13 [1.07; 1.19] | 0.00001 | 0.0001 | 1.09 [1.04; 1.14] | 0.0007 | 0.01 | 1.12 [1.05; 1.20] | 0.0006 | 0.02 |
| PCDF-12378 | 1.07 [1.01; 1.12] | 0.01 | 0.01 | 1.04 [1.00; 1.09] | 0.07 | 0.12 | 1.05 [0.99; 1.12] | 0.1 | 0.2 |
| PCDF-23478 | 1.11 [1.06; 1.17] | 0.00004 | 0.0003 | 1.08 [1.03; 1.13] | 0.001 | 0.01 | 1.09 [1.02; 1.17] | 0.007 | 0.05 |
| BjF | 1.08 [1.03; 1.14] | 0.003 | 0.008 | 1.05 [1.01; 1.10] | 0.03 | 0.08 | 1.07 [1.01; 1.15] | 0.03 | 0.11 |
| BkF | 1.08 [1.02; 1.13] | 0.005 | 0.01 | 1.05 [1.01; 1.10] | 0.03 | 0.08 | 1.06 [1.00; 1.13] | 0.06 | 0.16 |
| CHR | 1.09 [1.04; 1.15] | 0.0009 | 0.003 | 1.06 [1.01; 1.11] | 0.02 | 0.07 | 1.08 [1.01; 1.15] | 0.02 | 0.09 |
| CPP | 1.09 [1.03; 1.14] | 0.0009 | 0.003 | 1.07 [1.02; 1.12] | 0.003 | 0.02 | 1.06 [1.00; 1.13] | 0.05 | 0.14 |
| DbaeP | 1.09 [1.03; 1.16] | 0.003 | 0.008 | 1.04 [0.99; 1.09] | 0.2 | 0.21 | 1.09 [1.02; 1.18] | 0.02 | 0.09 |
| DBahA | 1.11 [1.05; 1.17] | 0.0002 | 0.001 | 1.07 [1.02; 1.12] | 0.005 | 0.03 | 1.10 [1.03; 1.18] | 0.006 | 0.05 |
| FA | 1.10 [1.05; 1.17] | 0.0003 | 0.001 | 1.06 [1.01; 1.11] | 0.02 | 0.07 | 1.08 [1.01; 1.16] | 0.02 | 0.09 |
| IP | 1.08 [1.03; 1.14] | 0.003 | 0.008 | 1.06 [1.01; 1.11] | 0.02 | 0.07 | 1.07 [1.00; 1.14] | 0.05 | 0.14 |
| PHE | 1.10 [1.04; 1.16] | 0.0005 | 0.002 | 1.04 [1.00; 1.09] | 0.07 | 0.12 | 1.08 [1.01; 1.16] | 0.02 | 0.09 |
| PY | 1.10 [1.05; 1.16] | 0.0003 | 0.001 | 1.06 [1.02; 1.12] | 0.009 | 0.04 | 1.07 [1.00; 1.14] | 0.05 | 0.14 |
| BaA | 1.09 [1.03; 1.15] | 0.001 | 0.003 | 1.05 [1.00; 1.10] | 0.04 | 0.10 | 1.08 [1.02; 1.16] | 0.02 | 0.09 |
| BaP | 1.11 [1.05; 1.17] | 0.0001 | 0.0006 | 1.07 [1.02; 1.12] | 0.007 | 0.04 | 1.10 [1.03; 1.17] | 0.006 | 0.05 |
| BbF | 1.08 [1.02; 1.13] | 0.004 | 0.009 | 1.05 [1.00; 1.10] | 0.03 | 0.08 | 1.07 [1.00; 1.14] | 0.04 | 0.13 |
| BcFL | 1.07 [1.02; 1.13] | 0.007 | 0.01 | 1.03 [0.98; 1.08] | 0.2 | 0.2 | 1.07 [1.00; 1.14] | 0.05 | 0.14 |
| BghiP | 1.07 [1.02; 1.13] | 0.006 | 0.01 | 1.05 [1.00; 1.10] | 0.03 | 0.08 | 1.05 [0.99; 1.12] | 0.1 | 0.20 |
| Asi | 1.07 [1.02; 1.13] | 0.006 | 0.01 | 1.05 [1.00; 1.10] | 0.05 | 0.11 | 1.07 [1.01; 1.15] | 0.03 | 0.11 |
| CrVI | 1.08 [1.02; 1.14] | 0.004 | 0.009 | 1.07 [1.02; 1.12] | 0.007 | 0.04 | 1.06 [1.00; 1.14] | 0.07 | 0.18 |
| Ga | 1.07 [1.03; 1.12] | 0.002 | 0.006 | 1.10 [1.01; 1.19] | 0.03 | 0.08 | 1.08 [1.02; 1.14] | 0.007 | 0.05 |
| Ge | 1.06 [1.01; 1.11] | 0.01 | 0.01 | 1.05 [1.00; 1.09] | 0.04 | 0.10 | 1.07 [1.01; 1.14] | 0.02 | 0.09 |
| HgI | 1.07 [1.02; 1.13] | 0.01 | 0.01 | 1.05 [1.00; 1.10] | 0.03 | 0.08 | 1.05 [0.99; 1.13] | 0.1 | 0.2 |
| Pb | 1.07 [1.01; 1.14] | 0.02 | 0.03 | 1.06 [1.00; 1.12] | 0.04 | 0.10 | 1.05 [0.98; 1.13] | 0.2 | 0.3 |
| Sb | 1.07 [1.02; 1.13] | 0.01 | 0.01 | 1.04 [0.99; 1.09] | 0.09 | 0.13 | 1.07 [1.00; 1.15] | 0.04 | 0.13 |
| Sn | 1.06 [1.01; 1.11] | 0.01 | 0.01 | 1.04 [1.00; 1.09] | 0.07 | 0.12 | 1.04 [0.98; 1.11] | 0.1 | 0.2 |
| Sr | 1.06 [1.01; 1.11] | 0.02 | 0.03 | 1.04 [1.00; 1.09] | 0.05 | 0.11 | 1.04 [0.98; 1.11] | 0.2 | 0.3 |
| Te | 1.12 [1.06; 1.17] | 0.00001 | 0.0001 | 1.08 [1.04; 1.13] | 0.0004 | 0.01 | 1.13 [1.06; 1.20] | 0.0001 | 0.01 |
| V | 1.07 [1.02; 1.13] | 0.006 | 0.01 | 1.05 [1.00; 1.10] | 0.04 | 0.10 | 1.07 [1.01; 1.15] | 0.03 | 0.11 |
| Ba | 1.07 [1.01; 1.14] | 0.02 | 0.03 | 1.06 [1.00; 1.11] | 0.05 | 0.11 | 1.05 [0.97; 1.13] | 0.2 | 0.3 |
| Cd | 1.07 [1.01; 1.13] | 0.03 | 0.04 | 1.03 [0.98; 1.09] | 0.2 | 0.21 | 1.05 [0.97; 1.13] | 0.2 | 0.3 |
| Co | 1.08 [1.02; 1.14] | 0.01 | 0.01 | 1.04 [0.99; 1.10] | 0.1 | 0.13 | 1.05 [0.98; 1.13] | 0.2 | 0.3 |
| CrIII | 1.12 [1.05; 1.18] | 0.0003 | 0.001 | 1.07 [1.02; 1.13] | 0.01 | 0.04 | 1.11 [1.03; 1.20] | 0.007 | 0.05 |
| DON | 1.05 [1.01; 1.11] | 0.03 | 0.04 | 1.05 [1.00; 1.09] | 0.04 | 0.10 | 1.04 [0.98; 1.10] | 0.2 | 0.3 |
| FB1 | 1.06 [1.01; 1.11] | 0.01 | 0.015 | 1.05 [1.00; 1.09] | 0.03 | 0.08 | 1.07 [1.01; 1.13] | 0.02 | 0.09 |
| FB2 | 1.05 [1.01; 1.10] | 0.02 | 0.03 | 1.04 [1.00; 1.08] | 0.06 | 0.11 | 1.06 [1.00; 1.12] | 0.03 | 0.11 |
| MAS | 1.06 [1.01; 1.10] | 0.02 | 0.03 | 1.03 [0.99; 1.07] | 0.1 | 0.13 | 1.04 [0.98; 1.10] | 0.2 | 0.3 |
| T2 | 1.08 [1.03; 1.14] | 0.002 | 0.006 | 1.07 [1.02; 1.12] | 0.005 | 0.03 | 1.07 [1.01; 1.14] | 0.03 | 0.11 |
| Zer | 1.10 [1.03; 1.18] | 0.004 | 0.009 | 1.09 [1.03; 1.16] | 0.004 | 0.03 | 1.08 [1.00; 1.18] | 0.06 | 0.16 |
| DON15 | 1.05 [1.01; 1.10] | 0.02 | 0.03 | 1.09 [1.01; 1.19] | 0.04 | 0.10 | 1.07 [1.01; 1.13] | 0.02 | 0.09 |
| PCB-156 | 1.07 [1.02; 1.13] | 0.006 | 0.012 | 1.05 [1.00; 1.09] | 0.05 | 0.11 | 1.05 [0.99; 1.12] | 0.1 | 0.2 |
| PCB-157 | 1.07 [1.02; 1.12] | 0.01 | 0.015 | 1.04 [1.00; 1.09] | 0.08 | 0.13 | 1.05 [0.98; 1.11] | 0.2 | 0.3 |
| PCB-167 | 1.07 [1.02; 1.12] | 0.01 | 0.015 | 1.04 [1.00; 1.09] | 0.07 | 0.12 | 1.05 [0.99; 1.12] | 0.1 | 0.2 |
| PCB-169 | 1.08 [1.03; 1.13] | 0.003 | 0.008 | 1.05 [1.01; 1.10] | 0.03 | 0.08 | 1.05 [0.99; 1.12] | 0.1 | 0.2 |
| PCB-180 | 1.07 [1.02; 1.12] | 0.009 | 0.015 | 1.04 [1.00; 1.09] | 0.07 | 0.12 | 1.05 [0.98; 1.11] | 0.2 | 0.3 |
| PCB-189 | 1.07 [1.02; 1.12] | 0.01 | 0.015 | 1.04 [1.00; 1.09] | 0.07 | 0.12 | 1.04 [0.98; 1.11] | 0.2 | 0.3 |
| PCB-28 | 1.07 [1.01; 1.12] | 0.01 | 0.015 | 1.04 [0.99; 1.09] | 0.1 | 0.13 | 1.05 [0.99; 1.12] | 0.1 | 0.2 |
| PCB-81 | 1.09 [1.04; 1.15] | 0.0006 | 0.002 | 1.06 [1.01; 1.10] | 0.02 | 0.07 | 1.08 [1.01; 1.15] | 0.02 | 0.09 |
| PCB-105 | 1.07 [1.02; 1.12] | 0.009 | 0.015 | 1.04 [1.00; 1.09] | 0.08 | 0.13 | 1.05 [0.99; 1.12] | 0.1 | 0.2 |
| PCB-114 | 1.07 [1.02; 1.13] | 0.007 | 0.013 | 1.04 [1.00; 1.09] | 0.07 | 0.12 | 1.05 [0.99; 1.12] | 0.1 | 0.2 |
| PCB-118 | 1.08 [1.02; 1.13] | 0.004 | 0.009 | 1.05 [1.00; 1.09] | 0.05 | 0.11 | 1.06 [0.99; 1.13] | 0.08 | 0.2 |
| PCB-126 | 1.09 [1.04; 1.15] | 0.0005 | 0.002 | 1.06 [1.02; 1.11] | 0.009 | 0.04 | 1.07 [1.01; 1.15] | 0.03 | 0.11 |
| PCB-138 | 1.07 [1.02; 1.13] | 0.007 | 0.01 | 1.04 [1.00; 1.09] | 0.06 | 0.11 | 1.05 [0.99; 1.12] | 0.1 | 0.2 |
| PCB-153 | 1.06 [1.01; 1.12] | 0.02 | 0.03 | 1.04 [0.99; 1.09] | 0.09 | 0.13 | 1.04 [0.98; 1.11] | 0.2 | 0.3 |
| Chlorpropham | 1.05 [1.01; 1.11] | 0.03 | 0.04 | 1.02 [0.98; 1.06] | 0.4 | 0.4 | 1.06 [1.00; 1.12] | 0.06 | 0.16 |
| Pirimiphos-methyl | 1.06 [1.01; 1.11] | 0.02 | 0.03 | 1.05 [1.01; 1.10] | 0.02 | 0.07 | 1.05 [0.98; 1.11] | 0.1 | 0.2 |

Odds ratios (OR) [95% CI] for a variation of one standard deviation (SD) were calculated using a logistic regression model with multiple imputations for each chemical separately and adjusted for maternal characteristics (ag, education level, migration status, employment, household income, maternal rural residence, maternal region of residence, number of older children in the household, smoking during pregnancy, diet quality), child and birth characteristics (sex, mode of delivery, family history of allergies), maternity size and study wave. Only chemicals association with eczema with a p-value <0.05 were tabulated, q-values were calculated using the false discovery rate (FDR) procedure; a q-value <0.1 is considered significant after multiple testing correction.


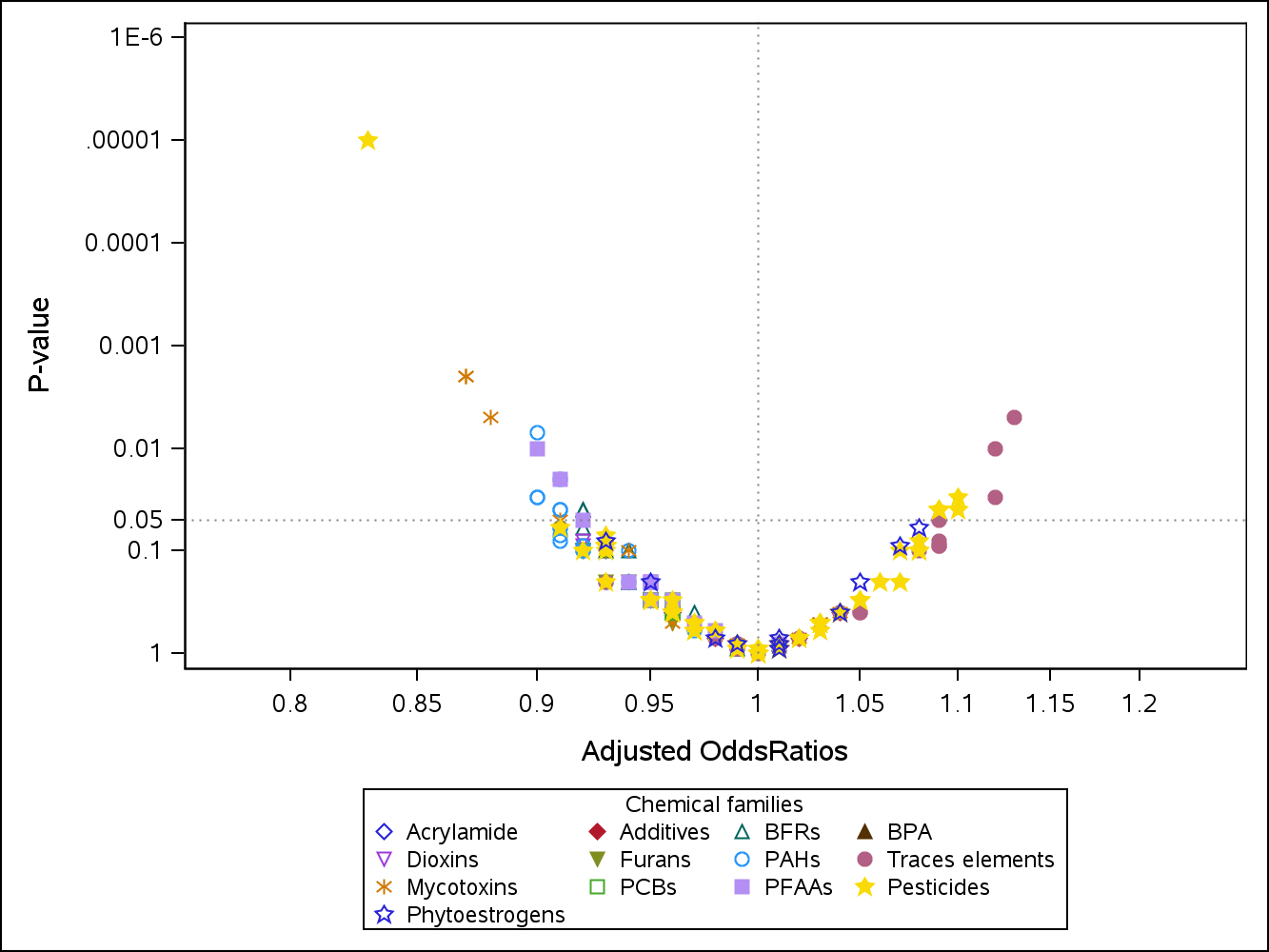


**Supplementary figure 3:** Associations between prenatal dietary exposure to individual chemicals and the risk of food allergies up to 5.5 y (n=11,635).

Odds ratios (OR) were calculated using a logistic regression model for each chemical separately and adjusted for maternal characteristics (age, education level, migration status, employment, household income, maternal rural residence, maternal region of residence, number of older children in the household, smoking during pregnancy, diet quality), child and birth characteristics (sex, mode of delivery, family history of allergies), maternity size and study wave. Prenatal exposure to food chemicals was log-transformed and then standardized. BPA: Bisphenol A; BFRs: Brominated flame retardants; PAHs: polycyclic aromatic hydrocarbons; PFAAs: perfluoroalkyl acids; PCBs: polychlorinated biphenyls.

**Supplementary table 5:** Associations between prenatal dietary exposure to individual chemicals and the risk of food allergy up to 5.5 y (with multiple testing correction)

|  | Multiple imputation  (n=11,635) | | | Complete case  (n=9,907) | | | Weighted analysis  (n=11,635) | | |
| --- | --- | --- | --- | --- | --- | --- | --- | --- | --- |
| Food chemical | **OR [95% CI]** | **p value** | **q value** | **OR [95% CI]** | **p value** | **q value** | **OR [95% CI]** | **p value** | **q value** |
| Li | 1.12 [1.03; 1.23] | 0.01 | 0.11 | 1.14 [1.05; 1.25] | 0.003 | 0.07 | 1.18 [1.06; 1.32] | 0.002 | 0.13 |
| Sb | 1.12 [1.01; 1.23] | 0.03 | 0.11 | 1.13 [1.02; 1.24] | 0.01 | 0.07 | 1.24 [1.10; 1.40] | 0.0006 | 0.08 |
| Sr | 1.13 [1.04; 1.24] | 0.005 | 0.09 | 1.13 [1.04; 1.23] | 0.004 | 0.07 | 1.13 [1.01; 1.27] | 0.03 | 0.3 |
| Chlorpyrifos-ethyl | 1.10 [1.01; 1.21] | 0.03 | 0.11 | 1.29 [1.08; 1.54] | 0.005 | 0.07 | 1.15 [1.02; 1.29] | 0.02 | 0.3 |
| BkF | 0.90 [0.81; 0.99] | 0.03 | 0.11 | 0.91 [0.83; 1.00] | 0.04 | 0.19 | 0.94 [0.82; 1.07] | 0.4 | 0.8 |
| DbaiP | 0.91 [0.84; 0.98] | 0.02 | 0.11 | 0.87 [0.74; 1.02] | 0.08 | 0.3 | 1.00 [0.90; 1.10] | 1 | 1 |
| PY | 0.90 [0.81; 0.99] | 0.03 | 0.11 | 0.93 [0.85; 1.03] | 0.2 | 0.3 | 0.94 [0.83; 1.07] | 0.4 | 0.8 |
| DbahP | 0.90 [0.83; 0.97] | 0.007 | 0.10 | 0.82 [0.70; 0.95] | 0.009 | 0.07 | 0.95 [0.86; 1.05] | 0.3 | 0.7 |
| DON | 0.87 [0.80; 0.95] | 0.002 | 0.06 | 0.89 [0.82; 0.97] | 0.007 | 0.07 | 0.90 [0.80; 1.01] | 0.07 | 0.5 |
| Niv | 0.88 [0.81; 0.96] | 0.005 | 0.09 | 0.89 [0.82; 0.97] | 0.008 | 0.07 | 0.89 [0.80; 1.00] | 0.04 | 0.3 |
| OTA | 0.87 [0.80; 0.95] | 0.002 | 0.06 | 0.90 [0.83; 0.97] | 0.01 | 0.07 | 0.87 [0.78; 0.96] | 0.009 | 0.3 |
| Carbaryl | 0.83 [0.77; 0.90] | 0.00001 | 0.0008 | 0.70 [0.60; 0.81] | <0.00001 | <0.00001 | 0.85 [0.77; 0.93] | 0.0008 | 0.08 |
| PFDA | 0.90 [0.83; 0.98] | 0.01 | 0.11 | 0.84 [0.72; 0.98] | 0.03 | 0.17 | 0.91 [0.83; 1.01] | 0.08 | 0.5 |
| PFDoA | 0.91 [0.84; 0.98] | 0.02 | 0.11 | 0.84 [0.72; 0.98] | 0.02 | 0.13 | 0.93 [0.84; 1.02] | 0.1 | 0.5 |

Odds ratios (OR) [95% CI] for a variation of one standard deviation (SD) were calculated using a logistic regression model with multiple imputations, for each chemical separately and adjusted for maternal characteristics (age, education level, migration status, employment, household income, maternal rural residence, maternal region of residence, number of older children in the household, smoking during pregnancy, diet quality), child and birth characteristics (sex, mode of delivery, family history of allergies), maternity size and study wave. Only chemicals association with food allergy with a p-value <0.05 were tabulated. q-values were calculated using the false discovery rate (FDR) procedure; a q-value <0.1 is considered significant after multiple testing correction.


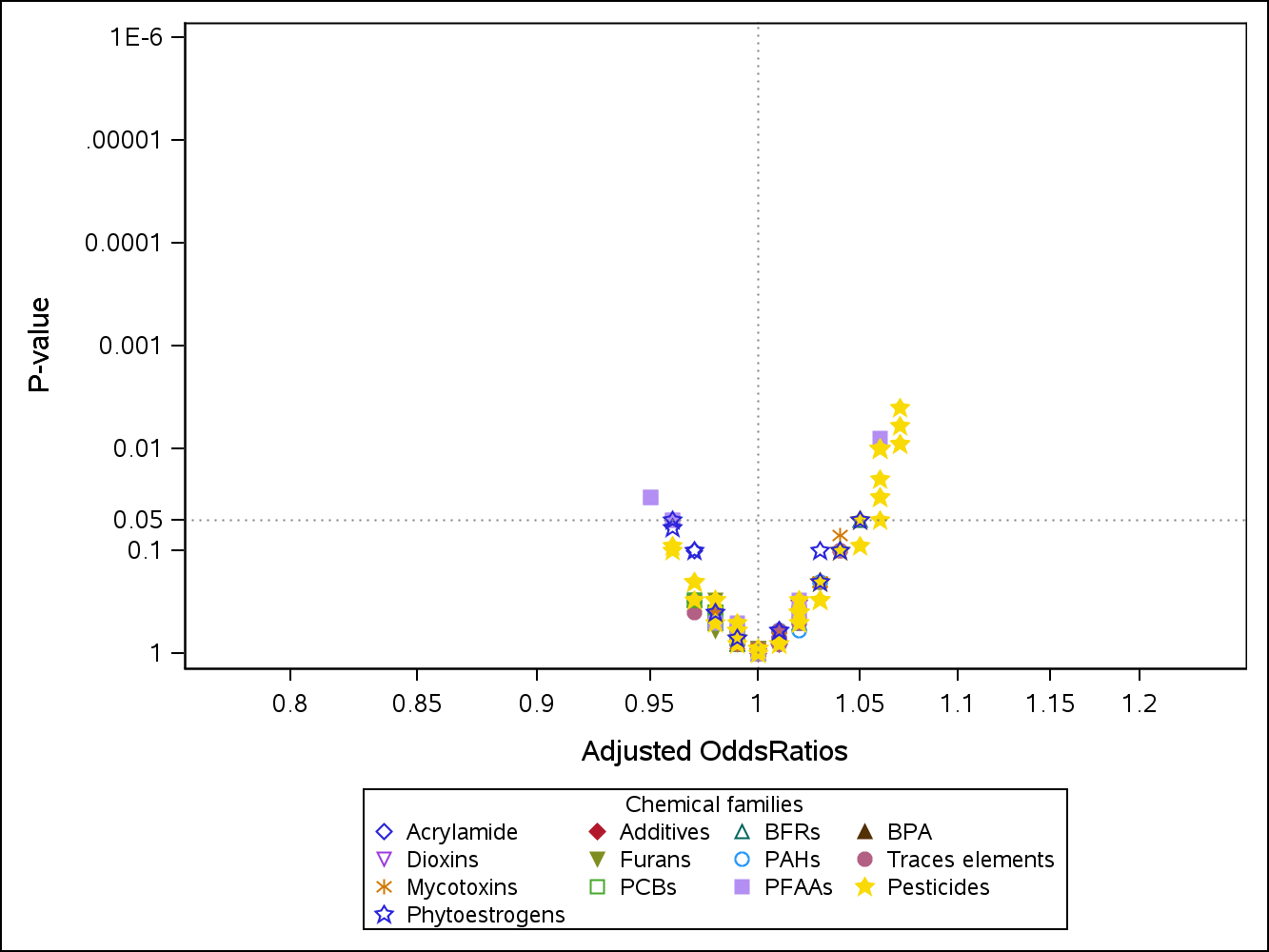


**Supplementary figure 4:** Associations between prenatal dietary exposure to individual chemicals and the risk of wheezing up to 5.5 y (n=11,636).

Odds ratios (OR) were calculated using a logistic regression model for each chemical separately and adjusted for maternal characteristics (age, education level, migration status, employment, household income, maternal rural residence, maternal region of residence, number of older children in the household, smoking during pregnancy, diet quality), child and birth characteristics (sex, mode of delivery, family history of allergies), maternity size and study wave. Prenatal exposure to food chemicals was log-transformed and then standardized. BPA: Bisphenol A; BFRs: Brominated flame retardants; PAHs: polycyclic aromatic hydrocarbons; PFAAs: perfluoroalkyl acids; PCBs: polychlorinated biphenyls.

**Supplementary table 6:** Associations between prenatal dietary exposure to individual chemicals and the risk of wheezing up to 5.5 y (with multiple testing correction)

|  | Multiple imputation  (n=11,636) | | | Complete case  (n=9,908) | | | Weighting analysis  (n=11,636) | | |
| --- | --- | --- | --- | --- | --- | --- | --- | --- | --- |
| Food chemical | **OR [95% CI]** | **p value** | **q value** | **OR [95% CI]** | **p value** | **q value** | **OR [95% CI]** | **p value** | **q value** |
| Boscalid | 1.07 [1.02; 1.12] | 0.009 | 0.13 | 0.97 [0.93; 1.02] | 0.2 | 0.3 | 1.03 [0.97; 1.09] | 0.4 | 0.6 |
| Fenhexamid | 1.07 [1.02; 1.13] | 0.004 | 0.13 | 1.06 [0.98; 1.15] | 0.2 | 0.3 | 1.08 [1.02; 1.15] | 0.01 | 0.3 |
| Fludioxonyl | 1.06 [1.01; 1.11] | 0.01 | 0.13 | 1.00 [0.96; 1.04] | 1 | 0.3 | 1.05 [0.99; 1.11] | 0.1 | 0.5 |
| Iprodione | 1.06 [1.01; 1.11] | 0.02 | 0.3 | 1.01 [0.97; 1.05] | 0.7 | 0.3 | 1.05 [0.99; 1.12] | 0.1 | 0.5 |
| Lambda-Cyhalothrin | 1.07 [1.02; 1.12] | 0.009 | 0.13 | 0.99 [0.96; 1.04] | 0.8 | 0.3 | 1.06 [1.00; 1.13] | 0.04 | 0.4 |
| Myclobutanil | 1.06 [1.01; 1.11] | 0.01 | 0.13 | 0.98 [0.94; 1.03] | 0.4 | 0.3 | 1.06 [1.00; 1.13] | 0.04 | 0.4 |
| Quinoxyfen | 1.06 [1.01; 1.12] | 0.01 | 0.13 | 1.00 [0.96; 1.05] | 0.9 | 0.3 | 1.08 [1.01; 1.14] | 0.02 | 0.3 |
| Spiroxamine | 1.06 [1.01; 1.12] | 0.01 | 0.13 | 1.00 [0.95; 1.04] | 0.9 | 0.3 | 1.08 [1.01; 1.14] | 0.02 | 0.3 |
| Tebufenpyrad | 1.06 [1.01; 1.12] | 0.01 | 0.13 | 1.00 [0.92; 1.09] | 1 | 0.3 | 1.08 [1.01; 1.14] | 0.02 | 0.3 |
| Tetraconazole | 1.06 [1.01; 1.12] | 0.01 | 0.13 | 0.98 [0.93; 1.02] | 0.3 | 0.3 | 1.08 [1.01; 1.14] | 0.02 | 0.3 |
| Triadimenol | 1.06 [1.01; 1.11] | 0.03 | 0.3 | 0.99 [0.94; 1.03] | 0.5 | 0.3 | 1.08 [1.01; 1.14] | 0.01 | 0.3 |
| Trifloxystrobin | 1.06 [1.01; 1.12] | 0.01 | 0.13 | 0.98 [0.93; 1.02] | 0.3 | 0.3 | 1.08 [1.01; 1.14] | 0.02 | 0.3 |
| Cyfluthrin | 1.06 [1.01; 1.12] | 0.01 | 0.13 | 1.01 [0.93; 1.10] | 0.9 | 0.3 | 1.08 [1.01; 1.14] | 0.02 | 0.3 |
| Etofenprox | 1.06 [1.01; 1.12] | 0.01 | 0.13 | 0.98 [0.94; 1.03] | 0.4 | 0.3 | 1.08 [1.01; 1.14] | 0.02 | 0.3 |
| Mepanipyrim | 1.07 [1.02; 1.12] | 0.006 | 0.13 | 0.97 [0.92; 1.02] | 0.2 | 0.3 | 1.07 [1.01; 1.14] | 0.03 | 0.4 |
| PFNA | 1.06 [1.02; 1.11] | 0.008 | 0.13 | 1.10 [1.01; 1.20] | 0.03 | 0.3 | 1.07 [1.01; 1.13] | 0.02 | 0.3 |
| PFHpA | 0.95 [0.91; 0.99] | 0.03 | 0.3 | 0.96 [0.89; 1.05] | 0.4 | 0.7 | 0.97 [0.91; 1.02] | 0.2 | 0.6 |

Odds ratios (OR) [95% CI] for a variation of one standard deviation (SD) were calculated using a logistic regression model with multiple imputations, for each chemical separately and adjusted for maternal characteristics (age, education level, migration status, employment, household income, maternal rural residence, maternal region of residence, number of older children in the household, smoking during pregnancy, diet quality), child and birth characteristics (sex, mode of delivery, family history of allergies), maternity size and study wave. Only chemicals association with wheezing with a p-value <0.05 were tabulated. q-values were calculated using the false discovery rate (FDR) procedure; a q-value <0.1 is considered significant after multiple testing correction.


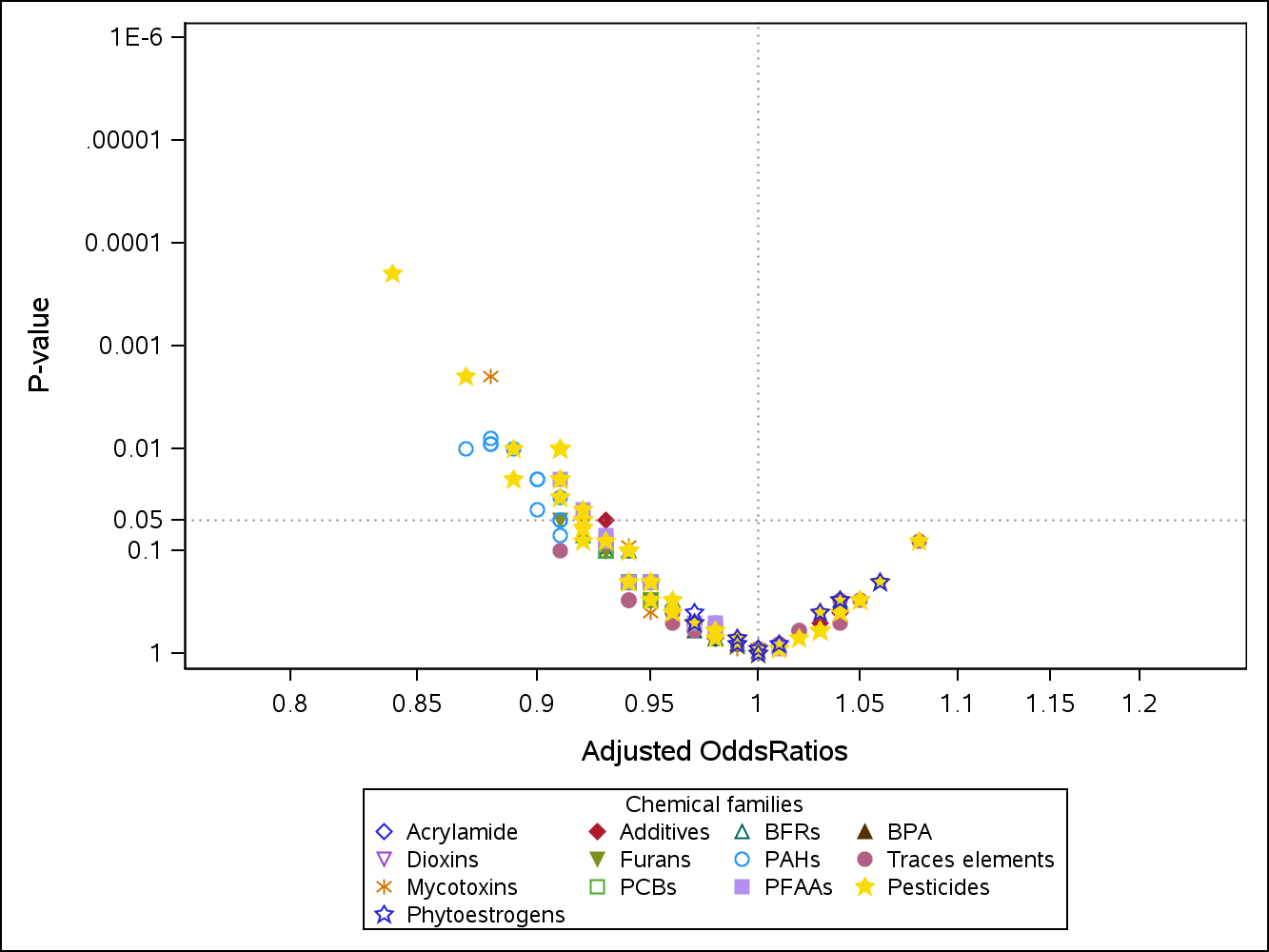


**Supplementary figure 5:** Associations between prenatal dietary exposure to individual chemicals and the risk of asthma up to 5.5 y (n=10,136).

Odds ratios (OR) were calculated using a logistic regression model for each chemical separately and adjusted for maternal characteristics (age, education level, migration status, employment, household income, maternal rural residence, maternal region of residence, number of older children in the household, smoking during pregnancy, diet quality), child and birth characteristics (sex, mode of delivery, family history of allergies), maternity size and study wave. Prenatal exposure to food chemicals was log-transformed and then standardized. BPA: Bisphenol A; BFRs: Brominated flame retardants; PAHs: polycyclic aromatic hydrocarbons; PFAAs: perfluoroalkyl acids; PCBs: polychlorinated biphenyls.

**Supplementary table 7:** Associations between prenatal dietary exposure to individual chemicals and the risk of asthma up to 5.5 y (with multiple testing correction)

|  | Multiple imputation  (n=10,136) | | | Complete case  (n=8,908) | | | Weighting analysis  (n=10,136) | | |
| --- | --- | --- | --- | --- | --- | --- | --- | --- | --- |
| Food chemical | **OR [95% CI]** | **p value** | **q value** | **OR [95% CI]** | **p value** | **q value** | **OR [95% CI]** | **p value** | **q value** |
| BjF | 0.88 [0.80; 0.97] | 0.009 | 0.06 | 0.90 [0.82; 0.99] | 0.03 | 0.12 | 0.88 [0.80; 0.97] | 0.009 | 0.11 |
| BkF | 0.88 [0.80; 0.97] | 0.008 | 0.06 | 0.90 [0.82; 0.99] | 0.03 | 0.12 | 0.87 [0.79; 0.96] | 0.005 | 0.11 |
| CHR | 0.89 [0.81; 0.97] | 0.01 | 0.06 | 0.91 [0.83; 1.00] | 0.05 | 0.15 | 0.88 [0.79; 0.97] | 0.01 | 0.11 |
| DbaeP | 0.87 [0.78; 0.97] | 0.01 | 0.06 | 0.89 [0.80; 0.99] | 0.04 | 0.14 | 0.88 [0.77; 0.99] | 0.04 | 0.11 |
| DBahA | 0.90 [0.82; 0.99] | 0.04 | 0.13 | 0.92 [0.84; 1.01] | 0.1 | 0.15 | 0.91 [0.81; 1.01] | 0.08 | 0.11 |
| IP | 0.91 [0.83; 0.99] | 0.03 | 0.11 | 0.93 [0.85; 1.02] | 0.1 | 0.15 | 0.91 [0.82; 1.00] | 0.05 | 0.11 |
| BaA | 0.90 [0.82; 0.98] | 0.02 | 0.08 | 0.91 [0.83; 1.00] | 0.05 | 0.15 | 0.91 [0.82; 1.01] | 0.06 | 0.11 |
| BbF | 0.88 [0.81; 0.97] | 0.009 | 0.06 | 0.90 [0.82; 0.99] | 0.03 | 0.12 | 0.87 [0.79; 0.96] | 0.005 | 0.11 |
| BcFL | 0.90 [0.82; 0.99] | 0.02 | 0.08 | 0.92 [0.84; 1.01] | 0.07 | 0.15 | 0.90 [0.81; 1.01] | 0.06 | 0.11 |
| DON15 | 0.88 [0.82; 0.95] | 0.002 | 0.06 | 0.77 [0.66; 0.90] | 0.001 | 0.04 | 0.88 [0.80; 0.97] | 0.009 | 0.11 |
| Triflumuron | 0.91 [0.84; 0.98] | 0.01 | 0.06 | 0.83 [0.71; 0.97] | 0.02 | 0.09 | 0.93 [0.85; 1.02] | 0.1 | 0.11 |
| Ethoxyquin | 0.91 [0.84; 0.98] | 0.01 | 0.06 | 0.83 [0.71; 0.97] | 0.02 | 0.09 | 0.93 [0.85; 1.02] | 0.1 | 0.11 |
| Azinphos-methyl | 0.91 [0.84; 0.98] | 0.01 | 0.06 | 0.83 [0.71; 0.97] | 0.02 | 0.09 | 0.93 [0.85; 1.02] | 0.1 | 0.11 |
| Captan | 0.91 [0.84; 0.98] | 0.01 | 0.06 | 0.83 [0.71; 0.97] | 0.02 | 0.09 | 0.93 [0.85; 1.02] | 0.1 | 0.11 |
| Diflubenzuron | 0.91 [0.84; 0.98] | 0.01 | 0.06 | 0.83 [0.71; 0.97] | 0.02 | 0.09 | 0.93 [0.85; 1.02] | 0.1 | 0.11 |
| Dimethoate | 0.87 [0.79; 0.95] | 0.002 | 0.06 | 0.76 [0.64; 0.91] | 0.003 | 0.07 | 0.93 [0.83; 1.05] | 0.2 | 0.2 |
| Fenbuconazole | 0.89 [0.81; 0.98] | 0.02 | 0.08 | 0.78 [0.64; 0.95] | 0.01 | 0.09 | 0.96 [0.85; 1.08] | 0.5 | 0.5 |
| Fludioxonyl | 0.91 [0.84; 0.99] | 0.03 | 0.11 | 0.93 [0.85; 1.01] | 0.08 | 0.15 | 0.94 [0.84; 1.06] | 0.3 | 0.3 |
| Iprodione | 0.89 [0.82; 0.97] | 0.01 | 0.06 | 0.91 [0.83; 0.99] | 0.02 | 0.09 | 0.94 [0.84; 1.05] | 0.2 | 0.2 |
| Phosalone | 0.91 [0.84; 0.99] | 0.03 | 0.11 | 0.86 [0.73; 1.02] | 0.08 | 0.15 | 0.93 [0.85; 1.03] | 0.2 | 0.2 |
| Phosmet | 0.91 [0.84; 0.99] | 0.02 | 0.08 | 0.79 [0.67; 0.93] | 0.005 | 0.09 | 0.93 [0.84; 1.03] | 0.2 | 0.2 |
| Procymidone | 0.91 [0.83; 0.99] | 0.02 | 0.08 | 0.81 [0.69; 0.96] | 0.02 | 0.09 | 0.92 [0.83; 1.02] | 0.1 | 0.11 |
| Propargite | 0.91 [0.84; 0.98] | 0.02 | 0.08 | 0.83 [0.70; 0.97] | 0.02 | 0.09 | 0.92 [0.84; 1.02] | 0.1 | 0.11 |
| Tebuconazole | 0.84 [0.77; 0.92] | 0.0002 | 0.02 | 0.70 [0.59; 0.84] | 0.0001 | 0.007 | 0.91 [0.81; 1.02] | 0.1 | 0.11 |
| Tebufenozid | 0.91 [0.84; 0.98] | 0.01 | 0.06 | 0.83 [0.71; 0.97] | 0.02 | 0.09 | 0.93 [0.85; 1.02] | 0.1 | 0.11 |
| Folpet | 0.91 [0.84; 0.98] | 0.01 | 0.06 | 0.83 [0.71; 0.97] | 0.02 | 0.09 | 0.93 [0.85; 1.02] | 0.1 | 0.11 |
| PFHpA | 0.91 [0.84; 0.98] | 0.02 | 0.08 | 0.88 [0.75; 1.04] | 0.1 | 0.15 | 0.92 [0.84; 1.02] | 0.1 | 0.11 |

Odds ratios (OR) [95% CI] for a variation of one standard deviation (SD) were calculated using a logistic regression model with multiple imputations, for each chemical separately and adjusted for maternal characteristics (age, education level, migration status, employment, household income, maternal rural residence, maternal region of residence, number of older children in the household, smoking during pregnancy, diet quality), child and birth characteristics (sex, mode of delivery, family history of allergies), maternity size and study wave. Only chemicals association with asthma with a p-value <0.05 were tabulated. Q-values were calculated using the false discovery rate (FDR) procedure; a Q-value <0.1 is considered significant after multiple testing correction.
